# Role of cystathionine-β-synthase and hydrogen sulfide in down syndrome

**DOI:** 10.1016/j.neurot.2025.e00584

**Published:** 2025-04-05

**Authors:** Csaba Szabo

**Affiliations:** Section of Pharmacology, Department of Oncology, Microbiology and Immunology, Faculty of Science and Medicine, University of Fribourg, Switzerland

**Keywords:** Hydrogen sulfide, Metabolism, Mitochondria, Trisomy, Bioenergetics, Transsulfuration

## Abstract

Down syndrome (DS) is a genetic condition where the person affected by it is born with an additional – full or partial – copy of chromosome 21. DS presents with characteristic morphological features and is associated with a wide range of biochemical alterations and maladaptations. Cystathionine-β-synthase (CBS) – one of the key mammalian enzymes responsible for the biogenesis of the gaseous transmitter hydrogen sulfide (H_2_S) – is located on chromosome 21, and people with DS exhibit a significant upregulation of this enzyme in their brain and other organs. Even though 3-mercaptopyruvate sulfurtransferase – another key mammalian enzyme responsible for the biogenesis of H_2_S and of reactive polysulfides – is not located on chromosome 21, there is also evidence for the upregulation of this enzyme in DS cells. The hypothesis that excess H_2_S in DS impairs mitochondrial function and cellular bioenergetics was first proposed in the 1990s and has been substantiated and expanded upon over the past 25 years. DS cells are in a state of metabolic suppression due to H_2_S-induced, reversible inhibition of mitochondrial Complex IV activity. The impairment of aerobic ATP generation in DS cells is partially compensated by an upregulation of glycolysis. The DS-associated metabolic impairment can be reversed by pharmacological CBS inhibition or CBS silencing. In rodent models of DS, CBS upregulation and H_2_S overproduction contribute to the development of cognitive dysfunction, alter brain electrical activity, and promote reactive gliosis: pharmacological inhibition or genetic correction of CBS overactivation reverses these alterations. CBS can be considered a preclinically validated drug target for the experimental therapy of DS.

## Introduction

In 1959 the French physicians Jerome Lejeune and Marthe Gautier discovered that DS has a genetic basis, consisting of an extra copy of chromosome 21 [1]. Lejeune subsequently proposed that the biochemical effects of the additional proteins encoded on the extra chromosome — including cystathionine-β-synthase (CBS) — may contribute to the biochemical alterations, metabolic maladaptations, and clinical manifestations of DS [2].

**Fig. 1:**
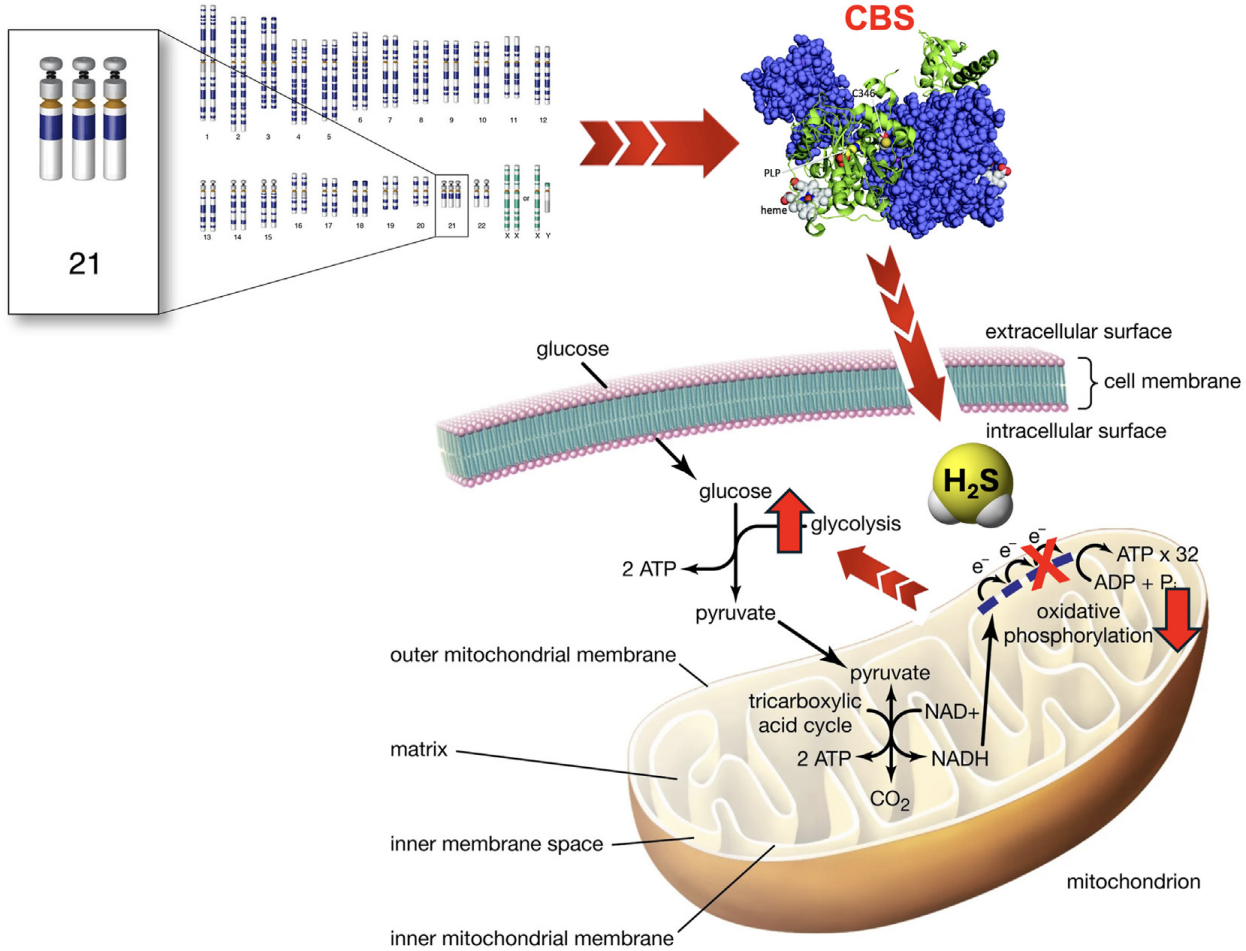
**The "Kamoun Hypothesis" in DS: overall visual summary**. The hypothesis posits that the overexpression of the CBS gene, due to its location on the triplicated chromosome 21 in DS, drives an abnormal increase in hydrogen sulfide (H_2_S) production, a signaling molecule that becomes neurotoxic at elevated levels. This excess H_2_S inhibits mitochondrial Complex IV (cytochrome *c* oxidase), disrupts mitochondrial function, impairs energy metabolism, and induces oxidative stress, collectively contributing to the cognitive deficits and neurodevelopmental delays characteristic of DS. As a compensatory response, DS cells activate alternative metabolic pathways such as glycolysis. By identifying the overactive CBS enzyme as a key driver of these effects, targeting CBS activity or neutralizing H_2_S could mitigate DS-associated neurological impairments.

In 1940's it was already noted that transsulfuration reactions can produce the gas hydrogen sulfide (H_2_S) in mammalian cells and tissues, but the functional role of mammalian H_2_S biogenesis was only investigated by a handful of biochemists until the 60's and 70's [3]. Mammalian H_2_S production came into a new focus in 1996, when Abe and Kimura demonstrated that CBS generates biologically relevant concentrations of H_2_S in the brain [[4], [5], [6]]. Inspired by this observation, and considering the well-known toxicological effects of H_2_S — whereby H_2_S exerts inhibits mitochondrial ATP generation by inhibiting mitochondrial Complex IV [7,8] — in the early 2000's Pierre Kamoun, a biochemist and physician colleague of Lejeune has put forward the hypothesis that CBS-derived overproduction of H_2_S may contribute to the pathogenesis of DS. Kamoun hypothesized that the excess H_2_S in DS inhibits cellular metabolism and adversely affects neurological development and central nervous system function [9,10] (Fig. 1). This hypothesis was supported by his clinical observations demonstrating that concentrations of the stable H_2_S metabolite thiosulfate and the H_2_S-hemoglobin reaction product sulfhemoglobin are increased in the urine and circulation of DS individuals [11,12]. The theory gained further support in 2005 when Kimura and colleagues demonstrated increased CBS expression in the brain of DS individuals [13].

**Fig. 2:**
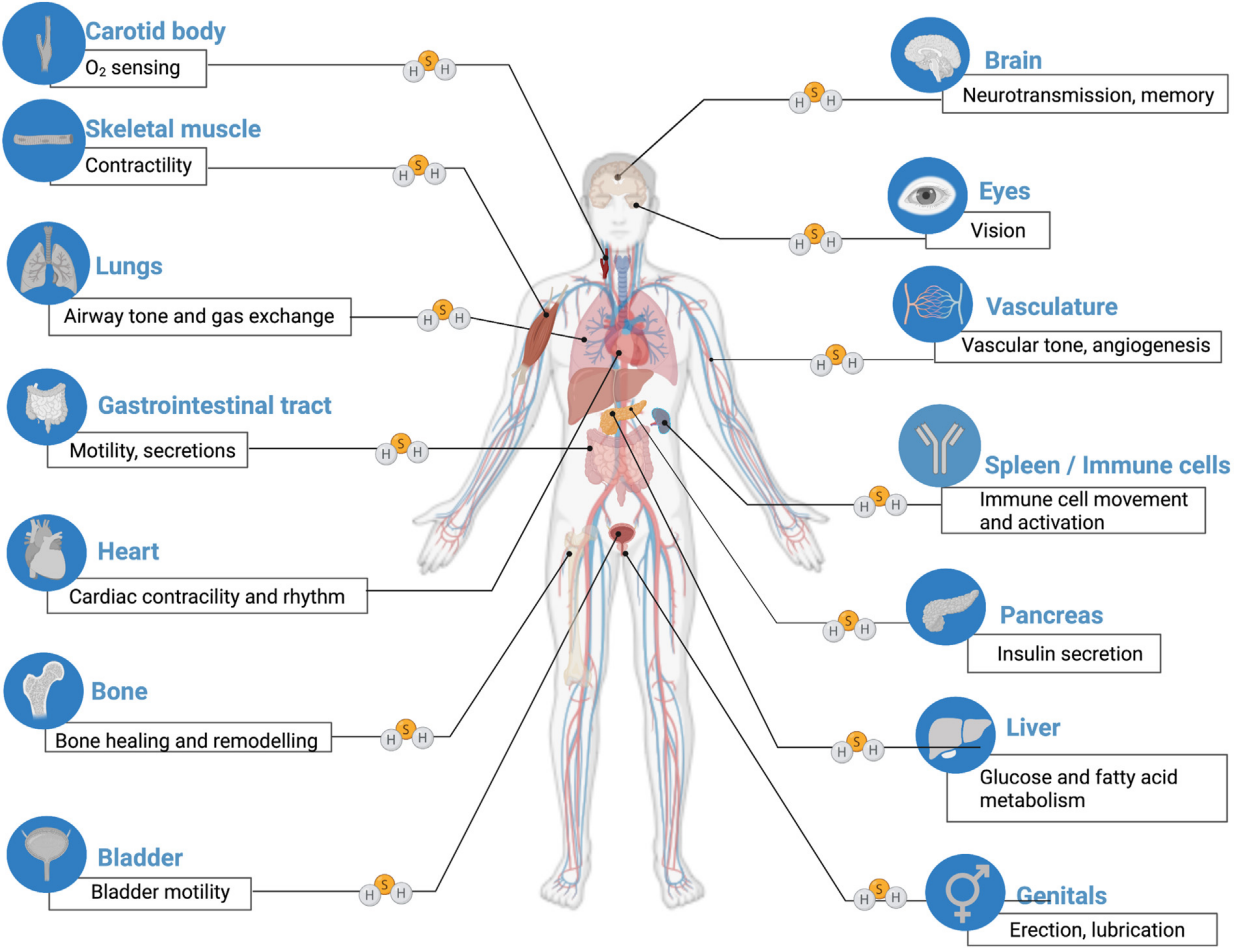
**Overview of the various physiological regulatory roles of endogenously produced H_2_S in the human body**. Reproduced from Ref. [17] by permission.

From the mid-2000's, investigations into the various biological regulatory roles of H_2_S have intensified, and H_2_S has emerged as the "third gasotransmitter" — alongside nitric oxide and carbon monoxide. Over the last two decades, thousands of papers have been published on the production and action of H_2_S in various physiological and pathophysiological conditions [[14], [15], [16], [17]]. H_2_S biogenesis, under normal physiological conditions, serves multiple regulator roles and purposes (Fig. 2), but there are also conditions where H_2_S levels are pathologically low, and others where H_2_S is overproduced and exerts pathophysiological effects [17]. As the field of H_2_S biology expanded, and as the methods to study cellular bioenergetics *in situ* improved over the last decade, the role of H_2_S and DS has been revisited using contemporary approaches. These recent studies [[18], [19], [20], [21], [22], [23], [24], [25], [26], [27], [28], [29], [30]] have confirmed and extended the Kamoun hypothesis, but also highlighted some of the complexities of the subject, and, in some cases, generated conflicting data. The current article reviews the state-of-the-art of this research field, reconciles the body of published literature and outlines the next steps necessary for the clinical translation of the concept.

## H_2_S overproduction in DS

H_2_S is a labile, reactive species, making its quantification in biological matrices challenging. Depending on the method used, attempts to measure H_2_S concentrations in the blood and plasma of humans and experimental animals have yielded divergent results. This challenge arises, at least, in part, from the existence of multiple reactive sulfur species in biological systems (including H_2_S, reactive sulfane sulfur species, and polysulfides), which are present in both free and bound 'pools' [[31], [32], [33], [34], [35]]. Nevertheless, it is generally accepted that existing detection methods, regardless of the technique used, are useful for comparing H_2_S levels between healthy controls and patient groups within the same study.

The first measurements related to H_2_S metabolites in DS were conducted by Kamoun's group over two decades ago [11,12]. In the first report, data were presented from 17 DS subjects, in comparison with healthy controls who were relatives of each DS subject (parent or, in some cases, sibling). Importantly, DS and control individuals followed the same diet, and sample collection was standardized, with urine samples collected in the morning as the first sample of the day. Thiosulfate, a stable degradation product of H_2_S, was then measured. It is also important to emphasize that in this study, urinary thiosulfate levels were normalized to the individual's glomerular filtration rate (GFR, assessed by the measurement of urinary creatinine concentration). In the DS group (17 subjects), thiosulfate/creatinine ratios were significantly higher than in the control group [11]. In the same study, urinary inorganic sulfate and cystine levels were also quantified; these analytes also tended to be higher in the DS samples, by approximately 10 ​% and 26 ​%, respectively [11].

**Fig. 3:**
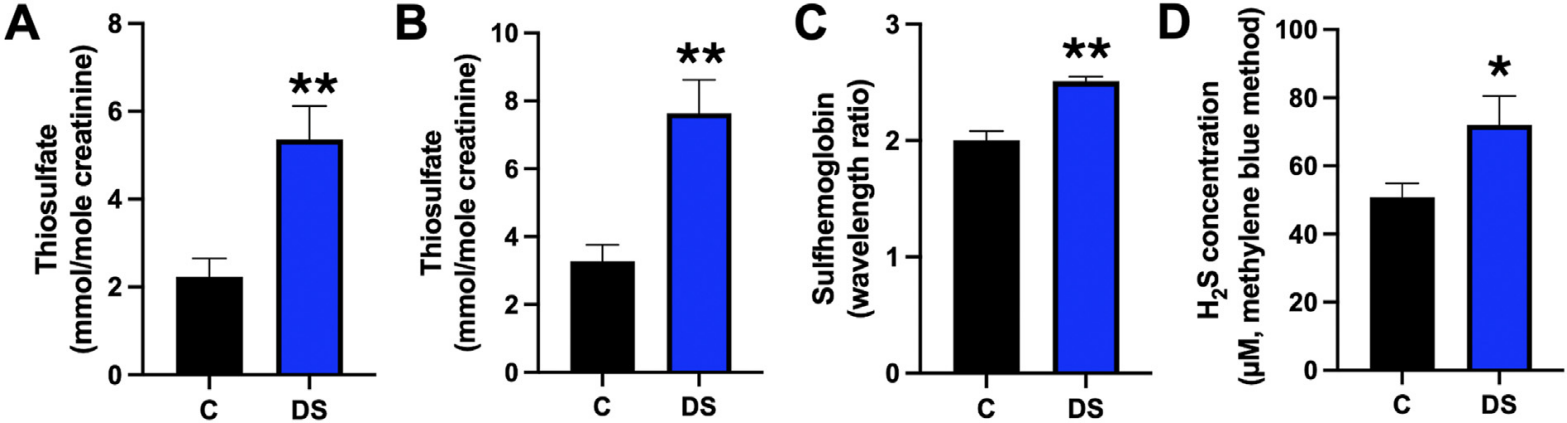
**Increased thiosulfate excretion, blood sulfhemoglobin content and blood H_2_S content in DS individuals. (A)**: Comparison of thiosulfate excretion in diet-matched pairs consisting of 21 pairs of subjects. In each volunteer family, one DS subject and one relative (mother or father in most families, brother or sister in rare cases) were given identical diets. This group consisted of 13 male and 8 female DS subjects and matched controls (10 male and 11 female). Sulfur compounds were excluded from the diet. The age of the control and DS groups in this comparison was 20 ​± ​2 vs. 51 ​± ​4 years, respectively. Analysis was performed from the first urine sample produced in the morning. **(B)**: Comparison of thiosulfate excretion in age-matched groups consisting of 30 DS subjects (19 male and 11 female) and 20 controls (volunteers from the laboratory; 10 male and 10 female). The age of the control and DS groups in this comparison was 20 ​± ​2 vs. 51 ​± ​4 years, respectively. Analysis was performed from the first urine sample produced in the morning. **(C)**: Comparison of blood sulfhemoglobin content, determined by spectrophotometry of 60 DS subjects (33 male and 27 female) and 60 age-matched normal controls (35 male and 25 female). The age of the control and DS groups in this comparison was 28 ​± ​8 vs. 27 ​± ​7 years, respectively. **(D)**: Comparison of blood H_2_S levels in age-matched DS and control subjects. 60 Down syndrome cases (33 males and 27 females, mean age 9 ​± ​4 years) were compared with 30 matched healthy normal children (18 males and 12 females mean age 9 ​± ​4 years). H_2_S levels were assessed in the plasma using the methylene blue method. Please note that this method is known to over-estimate the H_2_S levels [17]. The graphs shown in A-C represent a re-plotting of data published by Kamoun's group in 2003 [12], the graph shown in D represents a re-plotting of data published by Abdel-Salam and colleagues in 2003 [36].

In the second report published by the same group 2 years later [12] three groups of subjects were studied. The first group (21 DS and 21 control subjects, with mean age of 20 and 51 years, respectively) included the same 17 subjects already reported in their preceding paper, and utilized the identical experimental design as in the prior paper. Since these data are largely the same as in the prior paper, it is not surprising that the thiosulfate/creatinine ratios were also similar as in the prior report, with thiosulfate/creatinine levels over 2 times above the control values (Fig. 3A). In the second group, 30 DS subjects (19 male and 11 female) were compared to 20 age-matched, but unrelated controls (10 and 10 female volunteers from the investigators' laboratory). Once again, a significant difference was noted between healthy and DS individuals (Fig. 3B). In the third group of the same report, 60 DS subjects (33 male and 27 female) and 60 age-matched, but unrelated controls (35 male and 25 female) were analyzed for venous blood sulfhemoglobin levels: these levels were found approximately 25 ​% higher in DS individuals than in healthy controls (Fig. 3C) [12].

The next report investigating H_2_S levels in DS individuals was published 10 years later by a group working at the University of Cairo [36]. In this study, instead of urinary metabolite levels, blood levels of H_2_S were measured by the "methylene blue" method, which is based on the reaction of H_2_S with N,N-dimethyl-*p*-phenylenediamine dihydrochloride. This method is known to drastically overestimate the levels of H_2_S in the circulation [32], but – as mentioned earlier – it can be used to estimate relative differences in H_2_S levels between various groups. In this study, 60 DS children (33 males and 27 females, with mean age of 9 ​± ​4 years) were compared to 30 age-matched, but unrelated healthy normal children (18 males and 12 females). DS individuals were found to have H_2_S levels in their blood than in control (Fig. 3D) [36].

In contrast to the above findings, a recent paper reached a different conclusion. In a report by Antonaros and colleagues, published in 2024, 58 children with DS (23 females and 35 males, mean age 13 years, BMI 22) were compared to 48 controls (16 females and 32 males, mean age of 14 years) for various circulating H_2_S metabolites including urinary thiosulfate concentrations. Mean urinary thiosulfate concentrations in the DS group were reported to be 13 ​μM, while control values were approximately 20 ​% *higher* [37]. Importantly – and, in contrast to the Kamoun study – the urine samples were obtained from a biobank; no information was provided on the dietary aspects of the donors, no information was provided on the time of the day when the urine samples were obtained, and most importantly, the thiosulfate levels were not normalized to GFR (such as urinary creatinine). These factors all may have contributed to these findings.

Importantly, in a preceding publication from the same group, urinary concentrations of thiosulfate were analyzed in 8 volunteers. Each volunteer donated one morning sample over five consecutive days and urinary thiosulfate concentrations were expressed as μM – once again, without corrections for GFR. The mean thiosulfate concentrations in this study were reported as 15 ​± ​9 ​μM. But there were large variations in the values measured, with individual values ranging from 0.2 ​μM to 35 ​μM and mean values in individual volunteers ranging from approximately 3 ​μM–25 ​μM [38,39]. Also, when consecutive samples were obtained from the same healthy volunteer over a period of several days; the difference between the lowest and highest concentrations of thiosulfate measured in each volunteer was large: it amounted to 13 ​± ​8 ​μM. Clearly, measuring urinary thiosulfate levels without standardization (e.g., normalization to GFR) and without controlling for dietary and other factors introduces significant variability in urinary thiosulfate levels. This variability must be considered when interpreting findings or evaluating urinary thiosulfate as a potential biomarker for DS or other conditions.

The same group of authors, however, suggested that another urinary biomarker, trimethylsulfonium may have utility in the context of H_2_S and DS. Trimethylsulfonium is produced from H_2_S after conversion to methylthiol and then dimethylsulfide, and was reported to be higher in the urine of DS individuals than in the urine of healthy controls [37]. But this difference was only apparent after stratification of the subjects to "Trimethylsulfonium Producers" and ""Trimethylsulfonium Non-Producers" — based on a recently discovered polymorphism in the gene encoding for indole-ethylamine N-methyltransferase [40]. In this case, the latter subgroup was reported to have higher urinary trimethylsulfonium levels in DS (4.5 ​nM) than urinary trimethylsulfonium levels in healthy controls (3.1 ​nM) [37]. It should also be mentioned that urinary thiosulfate and urinary trimethylsulfonium levels did not show any correlation with each other. Moreover, urinary trimethylsulfonium levels exhibit high inter-individual as well diurnal variability [[39], [40], [41], [42]]. Thus, separation of the subjects to "Trimethylsulfonium Producers" and "Trimethylsulfonium Non-Producers" would likely represent a significant logistical challenges in future clinical studies.

In contrast to the above discussed, rather limited and somewhat conflicting clinical data, evidence for H_2_S overproduction in DS in cell-based models and animal models is substantial. In 2019, we have investigated the CBS/H_2_S pathway in human dermal fibroblasts from a control subject and an individual with DS (Detroit 551/ATCC CCL-110 and Detroit 539/ATCC CRL-84, respectively) and measured the cellular levels of H_2_S and reactive polysulfides using the H_2_S-sensitive dye 7-azido-4-methylcoumarin and the polysulfide-detecting Sulfane Sulfur Probe 4, respectively. Approximately 2.5-times higher H_2_S levels and more than 10-times higher polysulfide levels were measured in the DS cells than in the control cells [18]. In a follow-up study, 8 DS fibroblast cell lines and 8 healthy control cell lines were compared, and higher H_2_S levels — as well as higher reactive polysulfide levels — were confirmed in the DS cell cultures [21,25]. More recently, Derry and colleagues have compared H_2_S and polysulfide production from DS vs. healthy blood B lymphocytes (once again, using the AzMC and the SSP4 methods) and found approximately 2-fold higher H_2_S and 3-fold higher polysulfide levels in DS cells [28,29]. They have also demonstrated a strong correlation between elevated cellular H_2_S levels and the suppression of cell proliferation [29]. However, there was no correlation between CBS expression and cell proliferation in these DS cells, suggesting that – in addition to the upregulation of H_2_S producing enzymes — alterations in H_2_S catabolizing pathways also play important roles in determining the levels of H_2_S in DS [29] (see also below).

Data from animal studies related to H_2_S production and H_2_S levels in DS are currently rather limited. This limitation is primarily due to CBS being located on chromosome 17 in rodents, while most DS mouse models, for historical and technical reasons, do not include an extra copy of this chromosomal segment [[43], [44], [45], [46], [47]]. Only a handful of rodent DS models feature triplication of the chromosomal segment encoding CBS. Yann Herault's group investigated the functional role of the CBS pathway using the Dp(17Abcg1-Cbs)1Yah DS mouse model (commonly referred to as the Dp1Yah mouse) [19], but they did not report H_2_S levels or tissue H_2_S production rates. In a recently developed rat model of DS, which includes triplication of many — but not all — rat equivalents of genes on human chromosome 21, we measured H_2_S generation rates in homogenates from various brain regions, including the prefrontal cortex, hippocampus, entorhinal cortex, and basal forebrain. DS brains produced more H_2_S than control brains after incubation with cysteine and homocysteine, particularly in the prefrontal cortex and basal forebrain [24]. After the addition of the allosteric CBS activator S-adenosylmethionine (SAM), H_2_S production increased in the brain homogenates, and this response was most pronounced in the hippocampal region of the DS rats [24].

Most recently, using the Dp(17)3Yey/+ mice — another model of DS, which contains an extra copy of CBS, and several other genes encoded on mouse chromosome 17, but does not contain an extra copy of many other genes encoded on human chromosome 21 [48] — we higher H_2_S production rates were detected in the brain homogenates of DS mice than in control wild-type mice; this was associated with slightly elevated plasma (by 22 ​%) and brain (by 26 ​%) free H_2_S levels [27]. Unexpectedly, polysulfide levels in DS brains were *lower* than in wild-type controls. Protein persulfidation — a posttranslational modification induced by reactive polysulfides — was also *lower* in the DS brain samples than in control brains [27], highlighting the complex regulation and alterations of reactive sulfur species in this condition. One possible factor for this difference may be that — as discussed later — in DS not only H_2_S-generating enzymes, but also some of the H_2_S degradation enzymes, as well as other enzymes that can affect redox status and reactive oxygen species are dysregulated.

Taken together, the majority of the data discussed above are consistent with an overall increase in H_2_S levels in DS cells and DS animals, but there are outliers in the clinical body of literature regarding urinary thiosulfate levels in DS and there are some findings that are difficult to reconcile (i.e., higher H_2_S, but lower polysulfide levels in DS brains than in controls) in the mouse model.

## Regulation of H_2_S-producing and H_2_S-catabolizing enzymes in DS

**Fig. 4:**
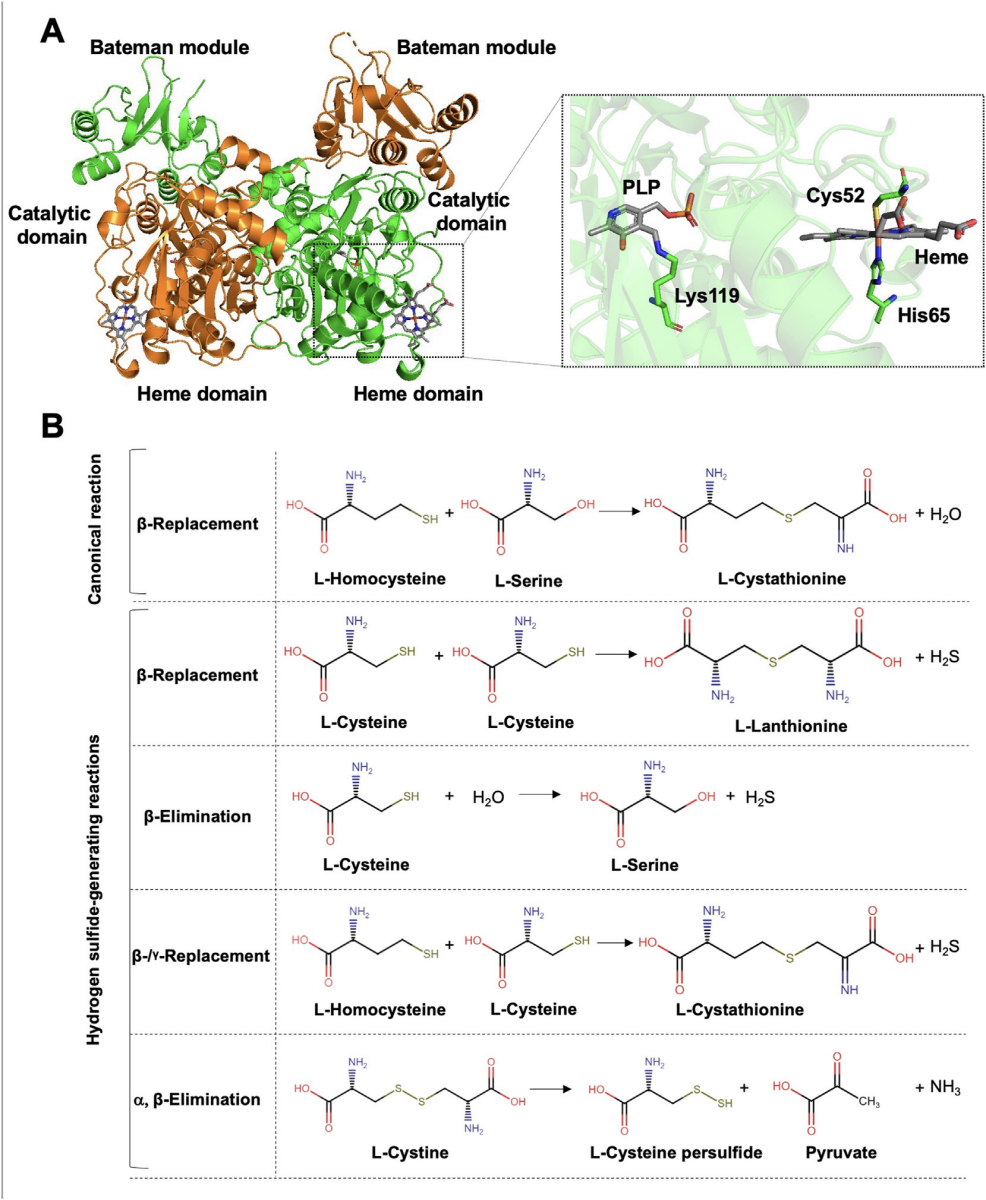
**Structure and function of CBS. (A)**: Crystal structure of the Δ516-525 human CBS homodimer (PDB# 4COO). Human CBS is architecturally organized in three regions: the Bateman module, the catalytic domain and the heme-binding domain. The engineered hCBS Δ516-525 is catalytically identical to the full-length native enzyme even if it lacks a loop consisting of 10 amino acid residues from the C-terminal regulatory domain. hCBS Δ516-525 forms dimers, rather than tetramers or higher order oligomers typical of the full-length CBS, that are colored in green and orange, respectively. The PLP and the heme cofactors are shown in sticks. The inset represents a zoom-in view into the catalytic (PLP) and regulatory (heme) sites. The PLP forms an internal aldimine intermediate via the Schiff base bond with the amino group of Lys 119, while the heme is coordinated by Cys 52 and His 65. (**B**): Scheme of the key biochemical reactions catalyzed by hCBS. Reproduced from Ref. [17] by permission.

**Fig. 5:**
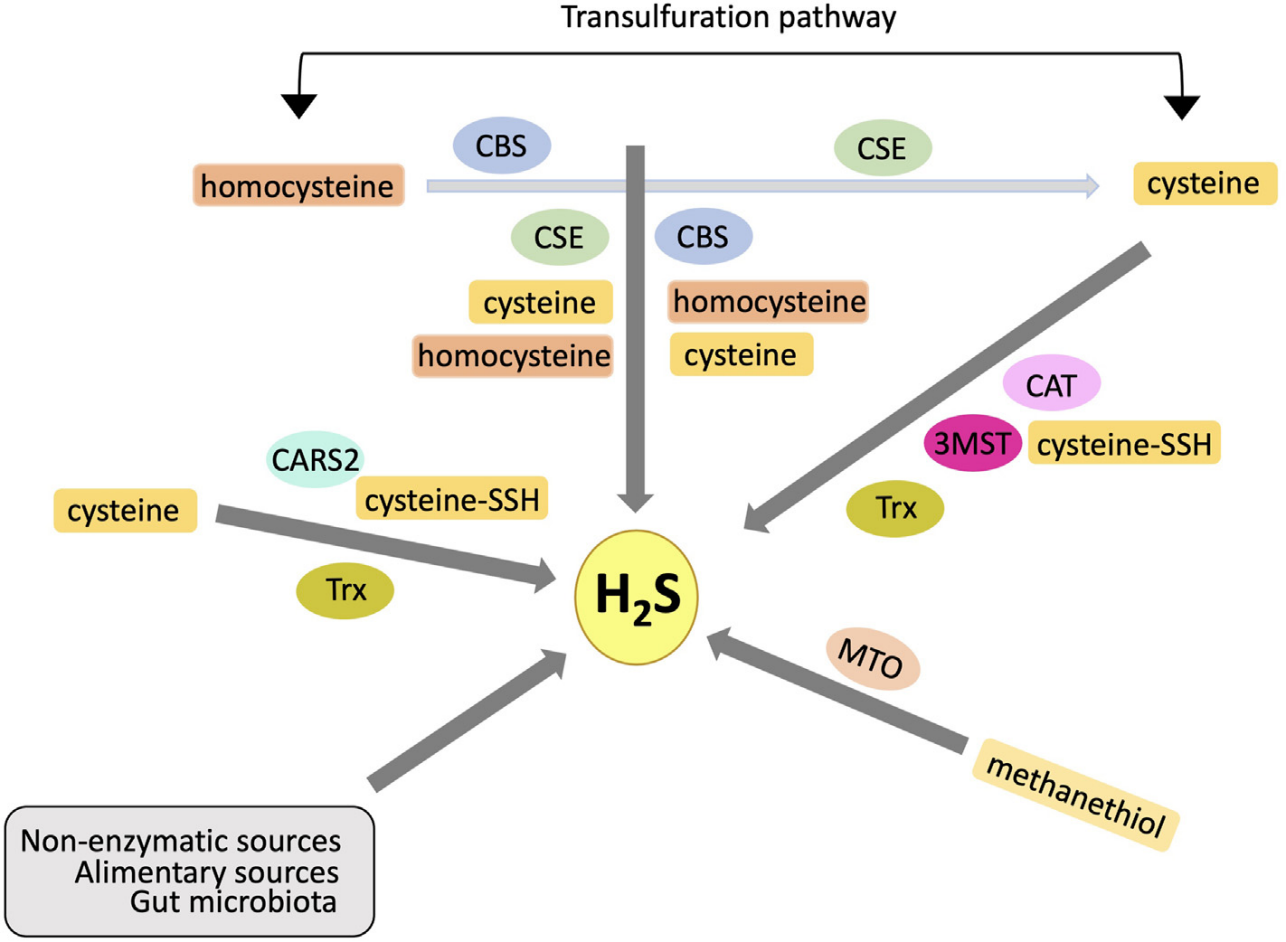
**Pathways of H_2_S generation in mammalian cells**. Cysteine and homocysteine serve as substrates for multiple reactions catalyzed by CSE and CBS to yield H_2_S. Cysteine is also catabolized via a cysteine aminotransferase (CAT)/3-MST pathway that leads to H_2_S production. In this case, H_2_S is liberated from persulfidated 3-MST through the action of thioredoxin (Trx) or reducing factors. Methanethiol is converted to H_2_S through the action of methanethiol oxidase (MTO). A recently described pathway that contributes to the levels of H_2_S involves the enzyme cysteinyl-tRNA synthetase 2 (CARS2) which forms cysteine persulfide (cysteine-SSH) that is incorporated in proteins. H_2_S is then released from persulfides through thioredoxins or other mechanisms. Non-enzymatic sources, gut microbiota and H_2_S-releasing molecules contained in the food also generate H_2_S and contribute to its endogenous levels. Reproduced from Ref. [17] by permission.

CBS, an important mammalian enzyme, is the first (and rate-limiting) enzyme in the transsulfuration pathway (Fig. 4). The biochemical character of CBS, its transcriptional and posttranscriptional regulation, its various biochemical roles as a transsulfuration enzyme, and pathophysiological states associated with CBS are subject to detailed, specialized review articles [17,49,50]. For the purpose of the current article, the most relevant aspects of CBS biochemistry are the following: **(a)** CBS, a homotetramer, with each monomer having a molecular weight of approximately 63 ​kDa, is ubiquitously expressed in many cells and tissues, with the brain, liver and kidney containing particularly high levels; **(b)** it is primarily cytosolic, although it can also be translocated into the mitochondria under certain conditions; **(c)** its physiological function lays in the transsulfuration pathway, where it links methionine metabolism to cysteine synthesis, but additional physiological functions have also been demonstrated in the brain due to its role in neural signaling via H_2_S; **(d)** one of its key enzymatic function is to catalyze the conversion of homocysteine and serine to cystathionine, which reaction also produces H_2_S, a gaseous signaling molecule involved in vasodilation, neuromodulation, and anti-inflammatory effects; **(e)** its catalytic activity is regulated by post-translational modifications, substrate availability, and interaction with cofactors, such as pyridoxal phosphate, which is an essential prosthetic group in its active site; **(f)** a key physiological regulatory mechanism of CBS activation is its allosteric modulation by S-adenosylmethionine (SAM); **(g)** CBS can also undergo proteolytic cleavage during inflammatory conditions or in response to oxidative stress, yielding a 45 ​kDa, constitutively activated truncated form of the enzyme. CBS is an essential part of the reverse transsulfuration pathway, and is one of the key enzymes responsible for mammalian H_2_S biogenesis [17]. The other major mammalian H_2_S-generating enzymes are cystathionine γ-lyase (CSE), 3-mercaptopyruvate sulfurtransferase (3-MST) and cysteinyl-tRNA synthetase 2 (CARS2) (Fig. 5) [17].

Since the gene for CBS is located on chromosome 21, the initial hypothesis was that CBS is upregulated in various cells and tissues in DS individuals due to a "gene dosage effect". The first direct evidence for elevated CBS levels in DS was provided by Chafedaux and colleagues in 1985 [51], who measured CBS activity (measurement of cystathionine production in cell homogenates in response to the addition of l-serine and l-homocysteine, a key reaction that CBS catalyzes, which, however, does not yield H_2_S, but, rather, produces cystathionine and water) in dermal fibroblasts from DS individuals with regular or partial chromosome 21 trisomies. In full trisomies, CBS activity was 66 ​% higher than control. In those partial trisomies that did not contain an extra segment of chromosome 17 that encodes for CBS, CBS activity was comparable to control; in fibroblasts from one DS subject whose partial trisomy contained the CBS coding region, CBS activity was 57 ​% higher than control [51]. The elevation in CBS activity was slightly higher than the expected gene dosage effect which predicted a 50 ​% increase in activity, but principally confirmed the gene dosage hypothesis, and was consistent with the known mapping of cbs to chromosome 21.

**Fig. 6:**
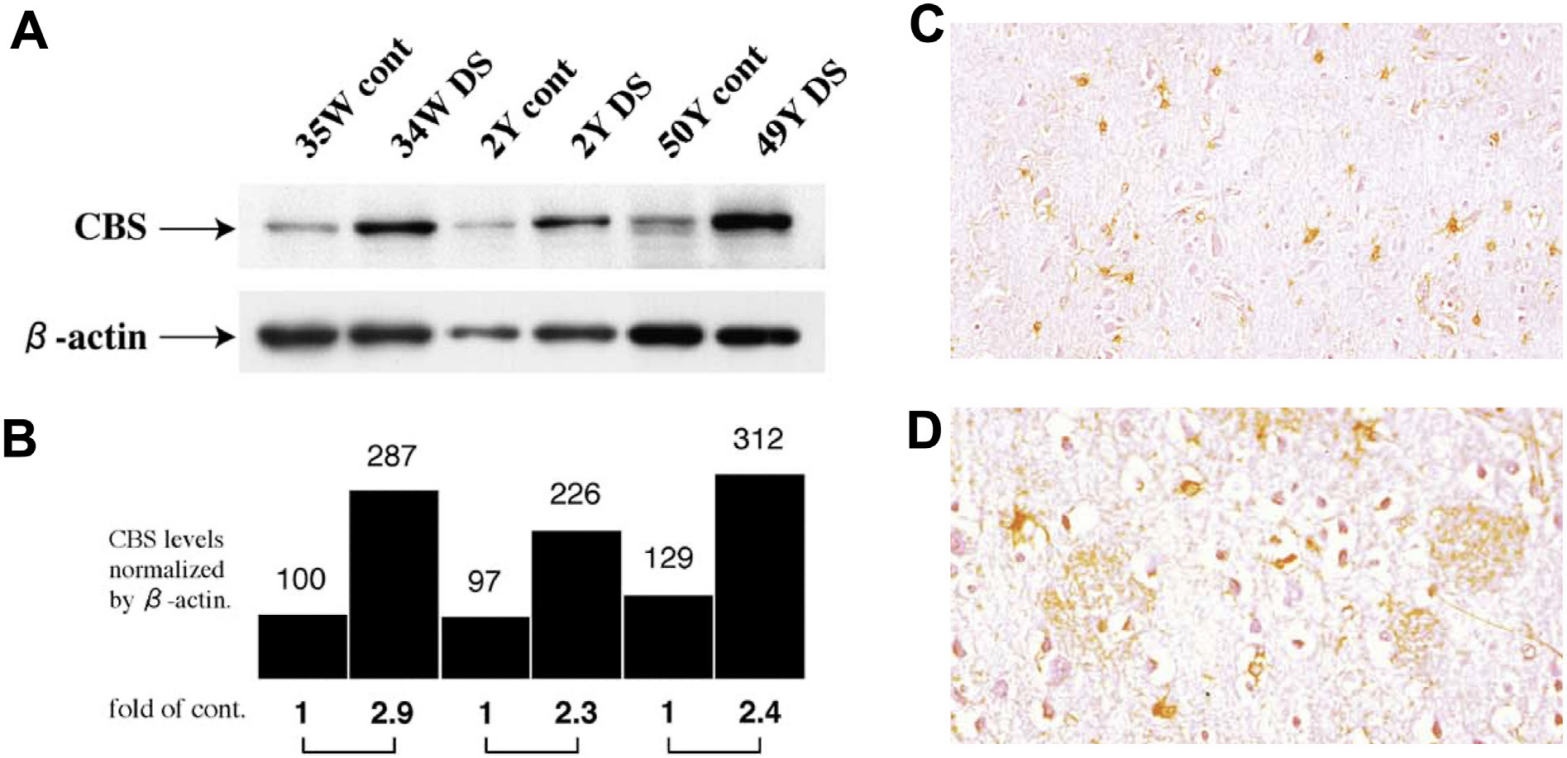
**Increased CBS protein in human DS brains**. Tissue samples were taken at autopsy, performed within 24 ​h postmortem, from the frontal lobes of DS individuals and normal controls from 34 weeks of gestation to 50 years of age. Western blot analysis was used to quantify the amount of CBS localized in the brain. (A): Samples were subjected to SDS–polyacrylamide gel electrophoresis and then transferred to nitrocellulose membrane. Western blot analysis was done with antibodies against CBS and beta-actin. **(B)**: Quantification of the amount of CBS localized in the brains of DS and normal individuals. The amounts of CBS are measured by densitometer and normalized with those of beta-actin. **(C)**: CBS in the cortex of brains of a 16 year old DS individuals' cerebral cortex primarily localizes to the astrocytes. **(D)**: Cerebral cortex of an adult DS individual with Alzheimer's disease: senile plaques and neurofibrillary tangles are present; astrocytes and microglia are associated with these structures and show CBS expression. Reproduced from Ref. [17] by permission.

Over the subsequent 30 years, several studies have confirmed and extended these observations, and demonstrated that human DS cells and tissues (Fig. 6a) [13] and tissues of DS rodents contain elevated levels of CBS mRNA, increased expression of CBS protein, and/or increased CBS enzymatic activity (Table 1) [13,18,23,24,27,29,[51], [52], [53], [54], [55], [56], [57], [58], [59], [60], [61], [62], [63], [64], [65], [66], [67], [68], [69]]. Most of the CBS in DS cells was cytosolic, but in DS — in contrast to the control cells – some of it also localized to the mitochondria [18]. Interestingly, in some experiments, not only the upregulation of total CBS protein, but also the emergence of the proteolytically activated, constitutively active 45 ​kDa isoform of CBS was observed; in the DS rat model, the truncated isoform was predominant in the prefrontal cortex and the basal forebrain [24]. Plasma and cellular levels of the allosteric CBS activator SAM are also elevated in human DS [70,71]. Thus, H_2_S generation in DS may not be simply related to a higher amount of CBS protein, but also to functional effects related to its differential allosteric or constitutive hyperactivation. Plasma levels of various metabolites — i.e., lower levels of homocysteine, methionine and serine and higher levels of cystathionine [22,23,[72], [73], [74], [75], [76], [77]] — are consistent with CBS overactivation.

**Table 1:** Studies investigating CBS expression or activity in DS cells and tissues.

DS Cell, tissue	Species	Method	Finding	First author, year	Reference
Dermal fibroblasts	Human	Enzymatic activity (cystathionine generation in cell homogenates)	DS cells have 66 ​% higher CBS activity than control cells	Chafedaux, 1985	[51]
Myeloblasts	Humans with AML or transient myeloproliferative disorder	PCR	DS blasts have over 10-times higher CBS mRNA levels	Taub, 1999	[52]
Fetal brain	Human	Western blotting	No difference noted between	Cheon, 2003	[53]
Brain tissue taken at autopsy	Human	Western blotting, immunohistochemistry	Approximately 3 times higher CBS in DS brains; mostly localized in astrocytes	Ichinohe, 2005	[13]
Cerebrum, cerebellum	Human, fetal	mRNA level (Affymetrix gene chip analysis)	DS tissues have higher CBS mRNA expression than controls; the effect is more pronounced in the cerebellum than in the cerebrum	Mao, 2005	[54]
Lymphoblastoid cells	Human	Dedicated oligonucleotide microarray	CBS mRNA in DS samples was 61 ​% higher than control	Aït Yahya-Graison, 2007	[55]
Cultured amniocytes and chorionic villus cells from pregnancies	Human	mRNA level (Affymetrix gene chip analysis)	Chorionic villus cells, but not amniocytes have higher CBS mRNA expression than controls	Altug-Teber et al., 2007	[56]
Fetal heart	Human	mRNA level (Affymetrix gene chip analysis)	No difference in CBS expression	Conti, 2007	[57]
Brain (temporal lobe and cerebellum)	Human	Immunohistochemistry	Higher CBS detected in DS, especially in the granular cell layers, primarily in the Bergmann glia cells.	Kanaumi, 2007	[58]
Prefrontal cortex tissue from a biobank	Human (fetal, adult)	mRNA level (Affymetrix gene chip analysis)	DS cortex has higher CBS mRNA expression than controls (40 ​% higher in fetal and 78 ​% higher in adult)	Lockstone, 2007	[59]
Corticomedullary sections of thymus	Human	mRNA level (Agilent microarray)	DS tissues have higher CBS mRNA expression than controls	Lima, 2011	[60]
Peripheral blood cells	Human (neonates and children separately analyzed)	mRNA level (Affymetrix gene chip analysis)	In peripheral blood cells from neonates, CBS mRNA expression was higher than control, but in peripheral blood mononuclear cells from children, no difference was found	Li, 2012	[61]
Induced pluripotent stem cells (iPSCs) from fetal cells	Human	mRNA level (Affymetrix gene chip analysis)	DS cells have higher CBS mRNA expression than controls	Chou, 2012	[62]
Induced pluripotent stem cells (iPSCs) from fetal cells and neurons generated from them	Human	mRNA level (Affymetrix gene chip analysis)	DS iPSCs have 2-times higher CBS mRNA expression than controls; DS neurons have 23 ​% higher expression than controls	Weick, 2013	[63]
Pluripotent stem cells derived from primary fetal skin fibroblasts	Human	RNA-seq (Illumina)	DS cells have higher CBS mRNA expression than controls	Letourneau et al., 2014	[64]
Dermal fibroblast cell lines	Human	RNA-seq (Illumina)	DS cells have higher CBS mRNA expression than controls	Sullivan, 2016	[65]
Peripheral blood cells	Human	RNA-seq (Illumina)	DS cells have higher CBS mRNA expression than controls	Sullivan, 2016	[65]
Choroid villus	Human	RNA-seq (Agilent)	DS cells have higher CBS mRNA expression than controls	Hervé, 2016	[66]
Fibroblast cell lines	Human	RNA-seq to assess mRNA expression and Pulsed SILAC & SWATH-MS to determine protein expression	No significant difference in CBS mRNA transcript, and variable increase in CBS protein	Liu et al., 2017	[67]
Fibroblast cell line Detroit 539	Human	Western blotting	DS cell line has 3.5 times higher CBS expression than controls	Panagaki, 2019	[18]
Peripheral blood leukocytes; plasma	Human (lean and obese)	RT-PCR	Obese DS subjects tend to have higher CBS expression than lean DS subjects	Meguid, 2022	[68]
Induced pluripotent stem cells (iPSC) lines	Human	Western blotting	DS cells tend to have higher CBS expression than controls	Susco, 2022	[69]
Fibroblast cell lines (n ​= ​8)	Human	Western blotting	DS cells have approximately 2.5 times higher CBS protein expression than controls, with significant subject-to-subject variability	Panagaki, 2022	[23]
Various brain regions	Rat (containing an extra copy of CBS and many additional genes equivalent to those present on human chromosome 21)	Western blotting	Full-length CBS protein is 50–100 ​% higher in various DS brain regions, while the 45 ​kDa truncated CBS is 2–4 fold higher. CBS activity in DS brain homogenates is significantly higher than in wild-type controls and is regionally different	Panagaki, 2022	[24]
Whole brain	Dp(17)3Yey/+ mouse (containing an extra copy of CBS and several additional genes encoded on human chromosome 21)	Western blotting	Full-length CBS protein is approximately 2-times higher in DS brain	Panagaki, 2024	[27]
Peripheral blood B lymphocytes	Human	Western blotting	Full-length CBS protein is approximately 3-times higher in DS cells	Mouli, 2024	[29]

The expression of CBS in various DS tissues exhibits cell-type selectivity: for example, in the DS brain, the higher expression of CBS is primarily localized to the astrocytes, as opposed to the neurons [13,24,27], and in adult human DS brains which also develop Alzheimer's type pathologies, CBS tends to concentrate in areas surrounding senile plaques and neurofibrillary tangles (Fig. 6) [13]. Regarding the relatively low levels of *neuronal* CBS overexpression in DS, there appears to be an age-dependence: in human cerebellum, CBS-positive neurons are detectable in the brain samples obtained in the fetal period and in infancy, but CBS expression is lower in brain samples obtained from adult DS individuals [13]. Indeed, a recent study, investigating CBS expression in healthy human populations also shows that CBS expression in the brain (both in whole brain samples and in astrocytes) shows an age-dependent decrease. At the level of mRNA, an approximately 90 ​% decrease is reported in the whole cortex and an approximately 50 ​% decrease in glia cells by age 60 [78]. At the protein level, the decrease is estimated to be approximately 50 ​% over the same time period [78].

**Fig. 7:**
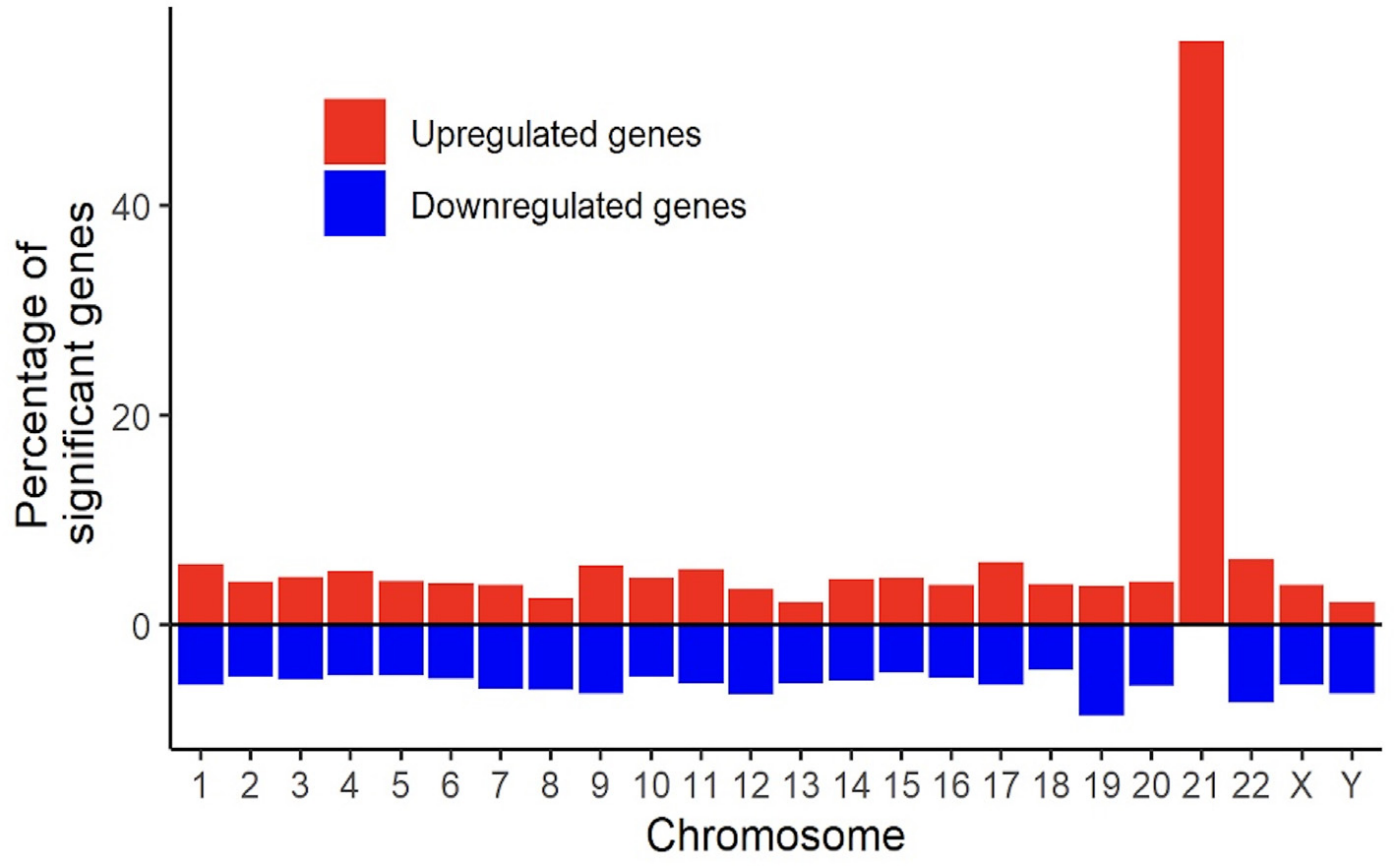
**Genome-wide changes in gene expression in DS**. Note that transcripts encoded on chromosome 21 show a uniform increase over control, while transcripts encoded on the other chromosomes show a wide-ranging changes, representing either increases or decreases in mRNA levels. Reproduced by permission from Ref. [22].

It should also be noted that there in some outlier reports CBS mRNA or protein was found *not* to be higher in DS samples than in controls. These studies include an early Western blot analysis of fetal brain samples [53] and an Affymetrix gene chip analysis of mRNA expression in human fetal heart samples [57]. The reason for this discrepancy may reflect small sample size, and/or methodological differences and/or heterogeneity in cell/tissue expression of various genes. For example, amniocytes from DS do not appear to have increased CBS expression (Table 1) [56]. It should also be emphasized that in some systems — e.g., in induced pluripotent stem cell systems [62,69] and when comparing various DS fibroblast lines [25] — the cell-line to cell-line (i.e. donor-to-donor) variability is rather high, perhaps reflecting individual differences in how various DS individuals' cells counterregulate and/or perhaps further amplify the gene dosage effect of the extra copy of cbs gene. Indeed, CBS protein levels in DS samples are often found to be *higher* than the 50 ​% increase that expected from a simple gene dosage effect (Table 1). It is possible that transcriptional processes regulating CBS expression [50,79] beyond the simple 'gene dosage effect' may also be contributing to the upregulation of CBS in DS. Indeed, when the global gene expression patterns in DS are investigated, it becomes obvious that a broad dysregulation of gene expression occurs with most of the genes are not encoded on chromosome 21; the majority of chromosome 21 genes are up-regulated, while on the other chromosomes both up- and downregulation of multiple genes is apparent, with thousands of genes affected in total (Fig. 7) [22,[55], [56], [57], [58], [59],^61^,^63^,^64^,^66^,^67^,[80], [81], [82], [83], [84]]. Furthermore, it is also clear that *mRNA expression data* and the expression data of the *corresponding protein* do not show a perfect correlation in DS samples [67,84].

**Fig. 8:**
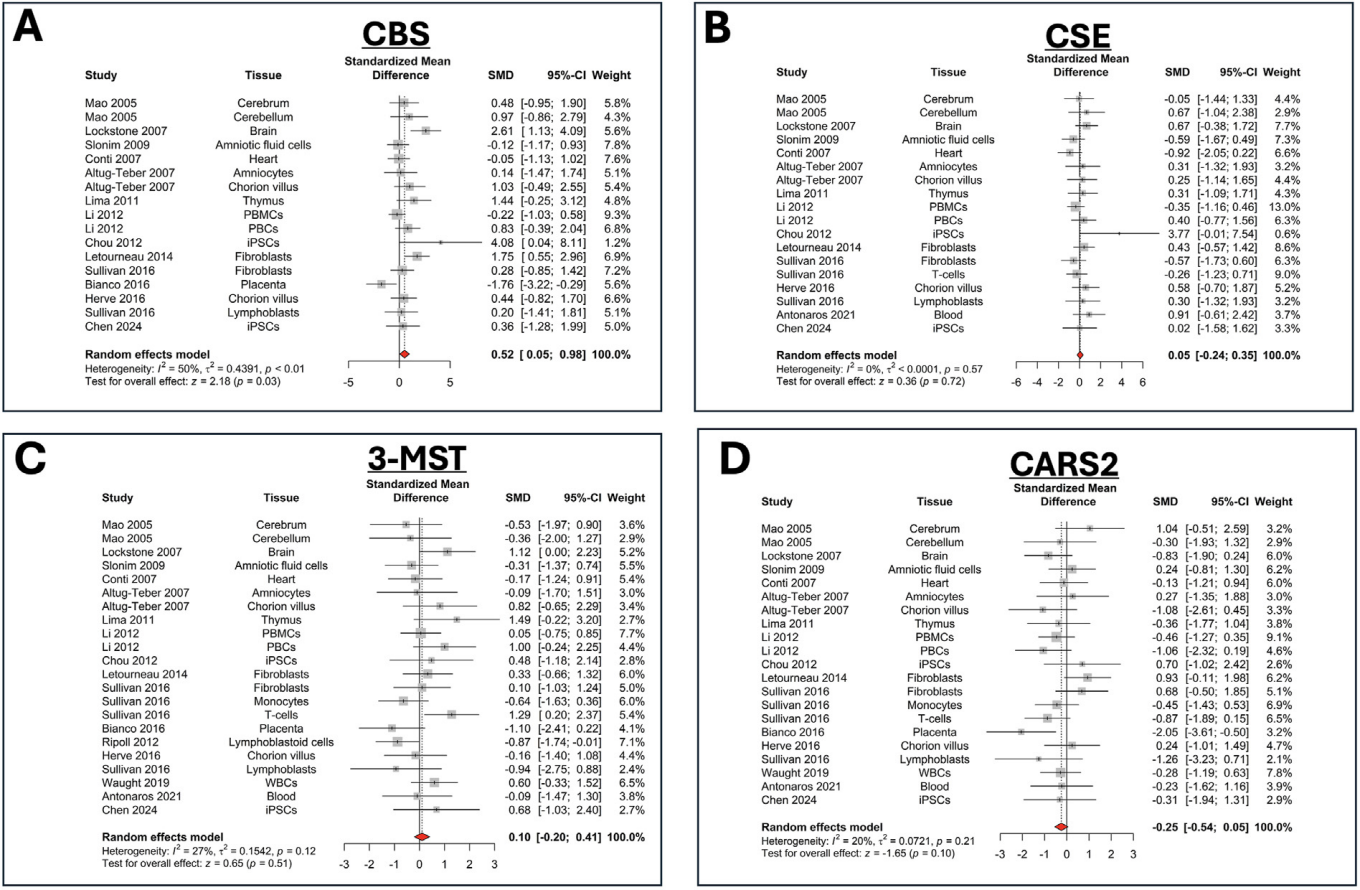
**Meta-analysis of the expression of the mRNA of the 4 principal H_2_S-producing enzymes CBS, CSE, 3-MST and CARS2 in human DS cells and tissues**. Forest plot showing relative weights, standardized mean difference (Hedge's g) with confidence intervals for CBS and PFKL gene expression. These genes are mapped on chromosome 21. Overall average effect size is displayed by ◆. The figures represent an updated analysis, using previously published methodology [22].

In DS fibroblast cell lines, there is also evidence for the upregulation of another important mammalian H_2_S-producing enzyme, 3-MST, as demonstrated by Western blotting. On average, from the analysis of 8 human DS fibroblast cell lines, DS is associated with an approximately 50 ​% higher 3-MST protein expression [21,25], with the majority of this increase localizing to the mitochondrial compartment [21]. CBS and 3-MST exhibit an inverse correlation across various DS fibroblast lines: DS cell lines that show the highest degree of CBS upregulation tend to exhibit relatively mild 3-MST upregulation, and vice versa (r ​= ​−0.6, p ​= ​0.06, n ​= ​8) [25]. In contrast to the changes in protein level, RNAseq comparison of various control vs. DS fibroblast lines does not show any overall difference in 3-MST mRNA expression in our meta-analysis (Fig. 8), although in better controlled comparisons — e.g., when 3-MST mRNA levels for a DS individual and the individual's twin are compared — the DS subject was found to present with an approximately 20 ​% higher 3-MST mRNA level than the control subject [67].

In animal studies, higher 3-MST expression was also noted in several (but not all) regions of the brain of the DS rat (an 50 ​% increase in the hippocampus, a 3-fold increase in the entorhinal cortex, and slight trend for an increase in the basal forebrain and no significant change in the prefrontal cortex) [24] and a very substantial, approximately 3-fold increase in the brain of the Dp(17)3Yey/+ mouse [27]. Interestingly, a micro-RNA target analysis conducted from thymic tissues of DS individuals vs. gender- and age-matched controls has identified several transcriptional modules in the DS samples, and one of these communities contained micro-RNAs that regulate mpst, the gene that encodes 3-MST [85].

As far as the regulation of the other two H_2_S-producing enzymes, cystathionine gamma-lyase (CSE) or cysteinyl-tRNA synthetase 2 (CARS2), meta-analysis of the available RNA sequencing databases of human cells and tissues does not show any significant effect in DS (Fig. 8). Likewise, no discernible differences were found in the expression of these enzymes between control vs. DS human cell lines or in rat or mouse models of DS [24,25,27]. Thus, it appears that these two enzymes are not directly relevant for the pathogenesis of DS.

**Fig. 9:**
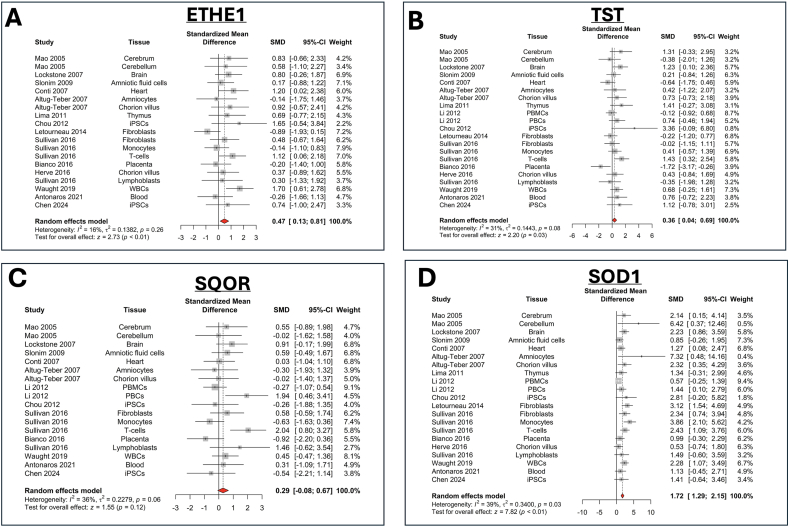
**Meta-analysis of the expression of the mRNA of key H_2_S-catabolizing enzymes ETHE1, TST (rhodanese), SQOR and SOD1 in human DS cells and tissues**. Forest plot showing relative weights, standardized mean difference (Hedge's g) with confidence intervals for CBS and PFKL gene expression. These genes are mapped on chromosome 21. Overall average effect size is displayed by ◆. The figures represent an updated analysis, using previously published methodology [22].

**Fig. 10:**
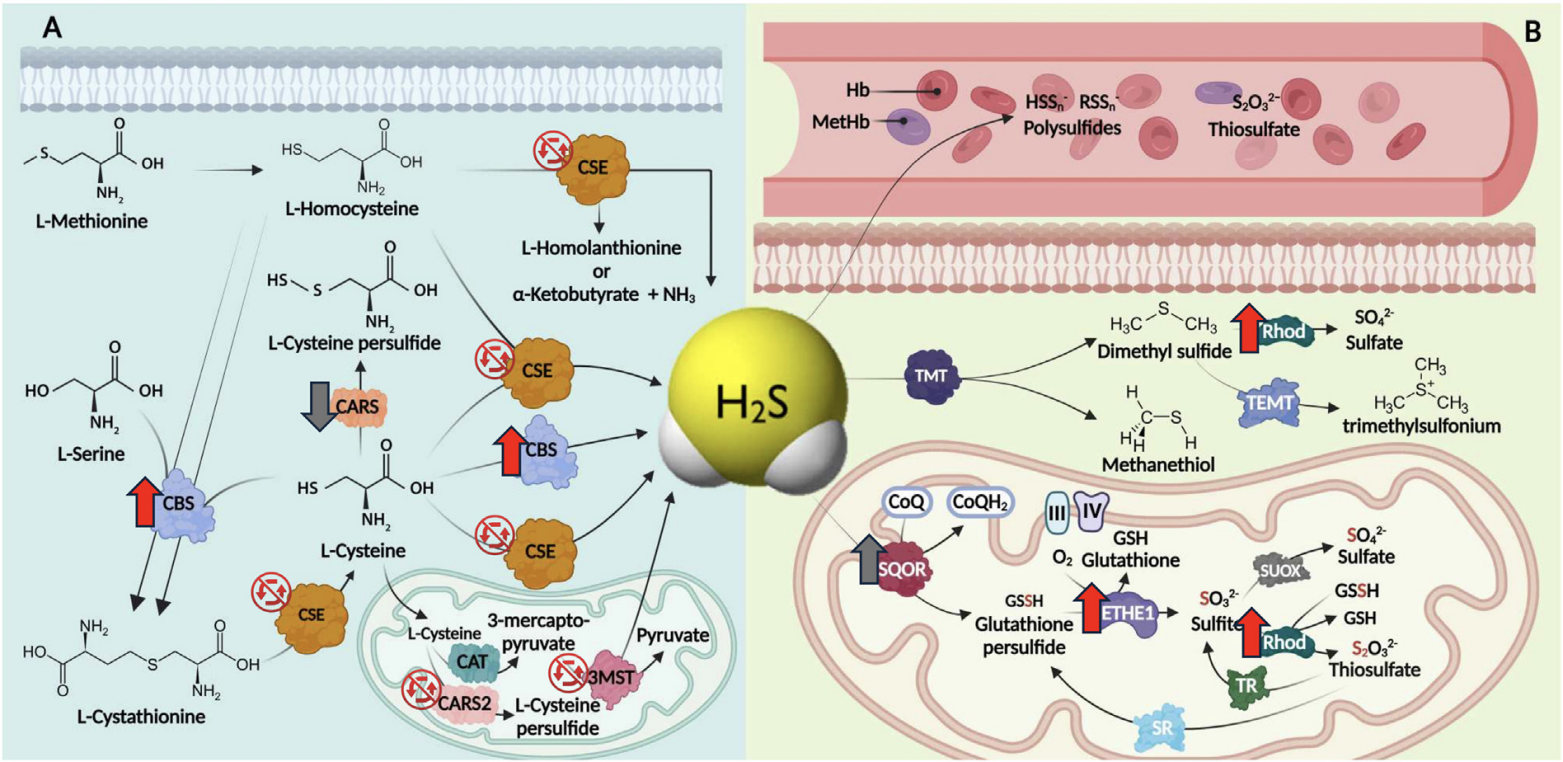
**Biosynthetic and degradation pathways of H_2_S; visual summary of the DS-associated alterations. (A)**: Anabolic pathways of H_2_S in the cytoplasm and mitochondrial matrix. **(B)**: Enzymatic and non-enzymatic catabolic pathways of H_2_S either through oxidation or methylation, where oxidation occurs in the mitochondria enzymatically or in the blood nonenzymatically. CBS: cystathionine β-synthase; CSE: cystathionine γ-lyase; 3-MST: 3-mercaptopyruvate sulfurtransferase; CARS: cysteinyl-tRNA synthetase 2; CAT: cysteine aminotransferase; SQOR: sulfide quinone oxidoreductase; ETHE1: ethylmalonic encephalopathy protein 1; Rhod: rhodanese; SUOX: sulfite oxidase; TR: thiosulfate reductase; SR: sulfur transferase; III: complex III; IV: complex IV; TMT: thiol S-methyltransferase; Hb: hemoglobin; MetHb: methemoglobin. Red arrows indicate DS-associated changes in the mRNA level of a given enzyme; gray arrows represent trends for a change; crossed out circular arrows represent no change. SOD1 (not shown in the scheme) is generally viewed as an antioxidant enzyme, but also has functions in H_2_S degradation. The mRNA of this enzyme is also significantly upregulated in DS. Please note that this analysis represents the changes on the mRNA levels; mRNA levels and proteins levels do not always or necessarily correlate with each other. The figure represents a modified version of a previously published scheme [16], reproduced by permission.

Interestingly, however, the upregulation of CBS and 3-MST in DS is sometimes associated with a parallel — likely compensatory — activation of H_2_S catabolism. In human DS fibroblasts, the H_2_S decomposition enzyme SQOR (but not ETHE1 or TST) shows a slight upregulation [24]; in the brain of the DS rat, in the prefrontal cortex (but not in other regions), TST and ETHE1 are upregulated [25], and the same pattern is also seen in the brain of the Dp(17)3Yey/+ mouse [27]. According to our meta-analysis of multiple human datasets, ETHE1, TST are significantly upregulated, and SQOR shows a tendency for upregulation (Fig. 9A–C). The antioxidant enzyme SOD1, encoded on chromosome 21, as expected, also shows an upregulation (Fig. 9D). Indeed, in a recent study, in B-cells from DS individuals, SOD1 expression was found to be upregulated, and functional studies using a SOD inhibitor demonstrated that this enzyme, indeed, plays a major role in downregulating free H_2_S levels (but upregulating polysulfide levels) [29]. The regulation of various H_2_S-generating and H_2_S-catabolizing enzymes in human DS — as assessed by a meta-analysis, which is based on mRNA expression (and not protein expression) data — is shown in Fig. 10. The simultaneous upregulation of H_2_S biosynthesis and H_2_S degradation in DS shows parallels with the situation in colon cancer cells: in epithelial cell organoids the sequential introduction of various cancer-associated mutations not only induces the upregulation of CBS and 3-MST, but also triggers the upregulation various H_2_S-decomposing enzymes, which partially compensate for the increased H_2_S production rate [86]. Similarly, in senescent macrophages, the simultaneous upregulation of multiple H_2_S-producing and H_2_S-degrading enzymes increases reactive sulfur turnover [87].

## Functional role of H_2_S in DS: *in vitro* studies

In addition to the progressive cognitive dysfunction [[88], [89], [90]], individuals with DS also exhibit significant systemic metabolic disturbances and decreased exercise tolerance [[91], [92], [93], [94]]. An important biomarker that signifies the metabolic alterations in DS individuals is the elevated circulating lactate [23,[95], [96], [97]], which suggests that DS tissues experience a hypoxia-like state and respond with a compensatory increase in glycolysis. In 2020 we have provided evidence for this phenomenon via a metabolomic metanalysis and proposed the term 'pseudohypoxia' [23].

The fact that DS cells exhibit suppressed mitochondrial function and have an overall impairment in their cellular bioenergetic function is well documented [[18], [19], [20], [21], [22], [23], [24],[98], [99], [100], [101], [102], [103], [104], [105], [106]]. The degree of mitochondrial suppression is dependent on the type of analysis: generally, methods that utilize whole-cell-based systems show a more pronounced effects than, for example, measurements conducted in isolated mitochondria. An interesting recent study utilized neurospheres derived from the embryos of Ts1Cje DS mouse model and observed significant maladjustments in multiple cellular metabolic pathways, further confirming an overall suppression of glucose utilization in DS, coupled with perturbations in the pentose phosphate pathway and a suppressed metabolism of glucose-6-phosphate [107]. Interestingly, neonatal astrocytes from the TS65Dn mice, which do *not* contain an extra copy of the cbs gene, do *not* exhibit suppressed cellular bioenergetic profile, but, instead, exhibit *increased* mitochondrial function [108]. In *in vivo* studies DS animals typically exhibit significant metabolic dysfunction [[109], [110], [111]]. Clearly, metabolic dysfunction in DS has a complex etiology and CBS overexpression and increased H_2_S generation cannot be the sole underlying pathogenetic factors. Nevertheless, increased H_2_S generation does appear to contribute to this response, as discussed in the following paragraphs.

One of the functional consequences of the impaired metabolic status of DS cells is the reduced proliferation and migration date, which has been well documented in multiple studies [18,25,79,[112], [113], [114], [115], [116], [117], [118], [119]]. Cell division and proliferation are ATP-dependent processes: impaired cellular bioenergetics in DS would, indeed, predict such functional consequences. In fact, when cells are exposed to various cytotoxic substances (H_2_S, but also cyanide) which decrease mitochondrial respiration via inhibition mitochondrial Complex IV, cellular ATP levels are decreased and cell proliferation is inhibited [7,8,16,17,120].

As mentioned above, DS cells tend to switch over to glycolysis as a compensatory response. Plasma metabolomics of DS individuals – showing increased lactate and pyruvate levels – confirm this phenomenon [94]. The switch has also been demonstrated in DS fibroblast cell lines [25] and in DS neural spheroids [107,121]. DS cells may also mobilize additional compensatory mechanisms, e.g., a preferential utilization of various non-traditional substrates such as glucose-1-phosphate (an intermediate in the breakdown of glycogen). Moreover, a comprehensive fluxomics analysis in human DS fibroblasts revealed that DS is associated with a significant increase in carbon fluxes from glucose into ribose/ribulose-5-phosphate and sedoheptulose 7-phosphate, suggesting the upregulation of the pentose-phosphate pathway [25]. This pathway may serve as an alternative pathway of NADH generation in DS cells, to partially compensate for the impaired activity of the Krebs cycle. (It should be noted that a similar metabolic rearrangement also occurs in cancer cells in response to CBS upregulation [122].) Finally, according to the findings of the fluxomics study, CBS inhibition also increases the carbon flux from glucose into glutamate [25]. This effect may also be relevant for the pathobiology of DS, because glutamate is an important as a metabolic factor, and its depletion was previously implicated in the pathogenesis of DS [[123], [124], [125]].

**Fig. 11:**
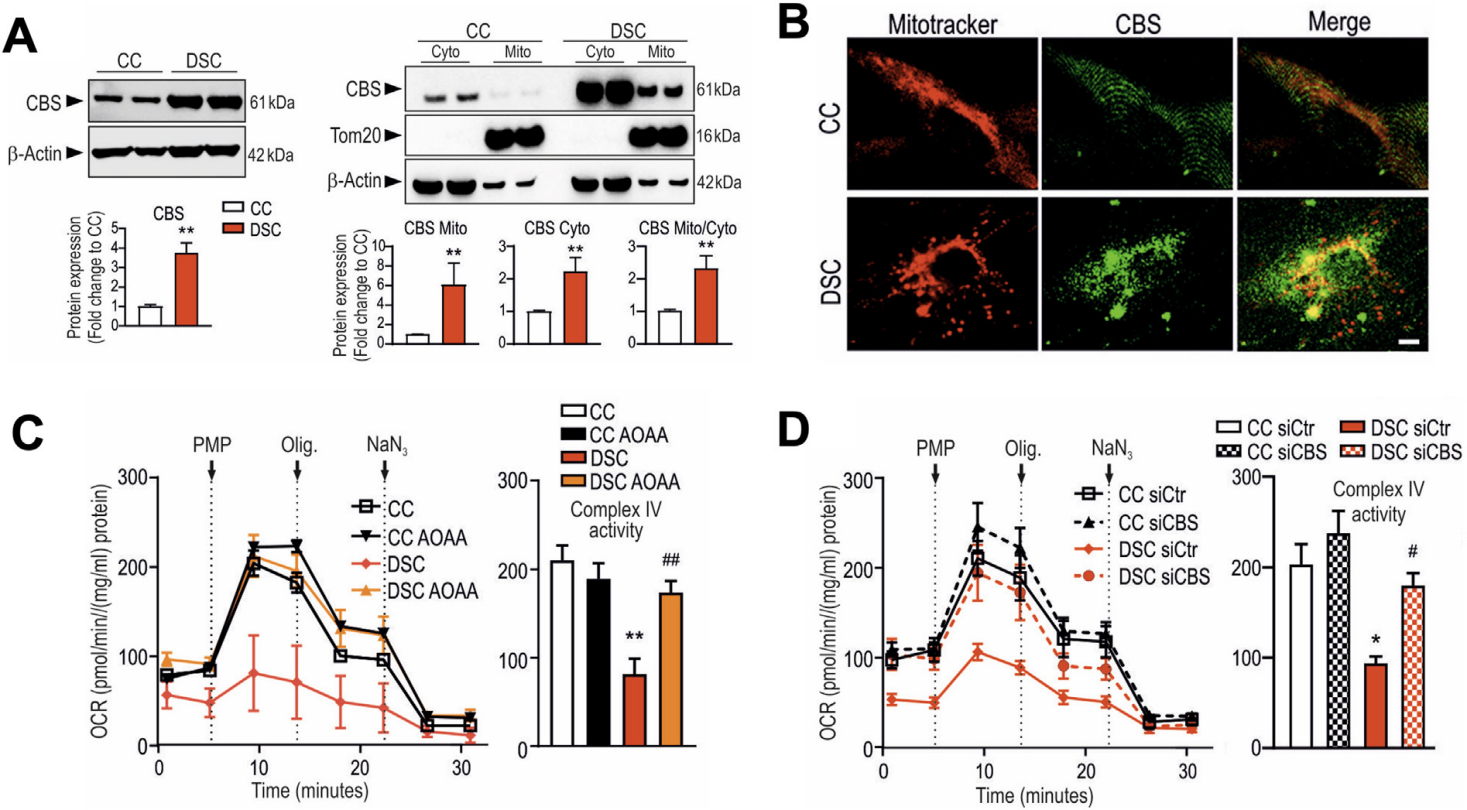
**CBS-derived H_2_S is responsible for the suppression of mitochondrial function in DS fibroblasts. (A)**: DS cells (DSC) exhibit markedly higher CBS expression than healthy control cells (CC) – which is, in part, localized to the mitochondria, as shown **(A)** by Western blotting and **(B)**: by confocal imaging. **(C**–**D)**: Complex IV is blocked in DS cells; Complex IV activity it is restored **(C)** by treatment of the cells with the CBS inhibitor AOAA or **(D)** by siRNA-mediated suppression of CBS expression. Reproduced by permission from Ref. [18].

**Fig. 12:**
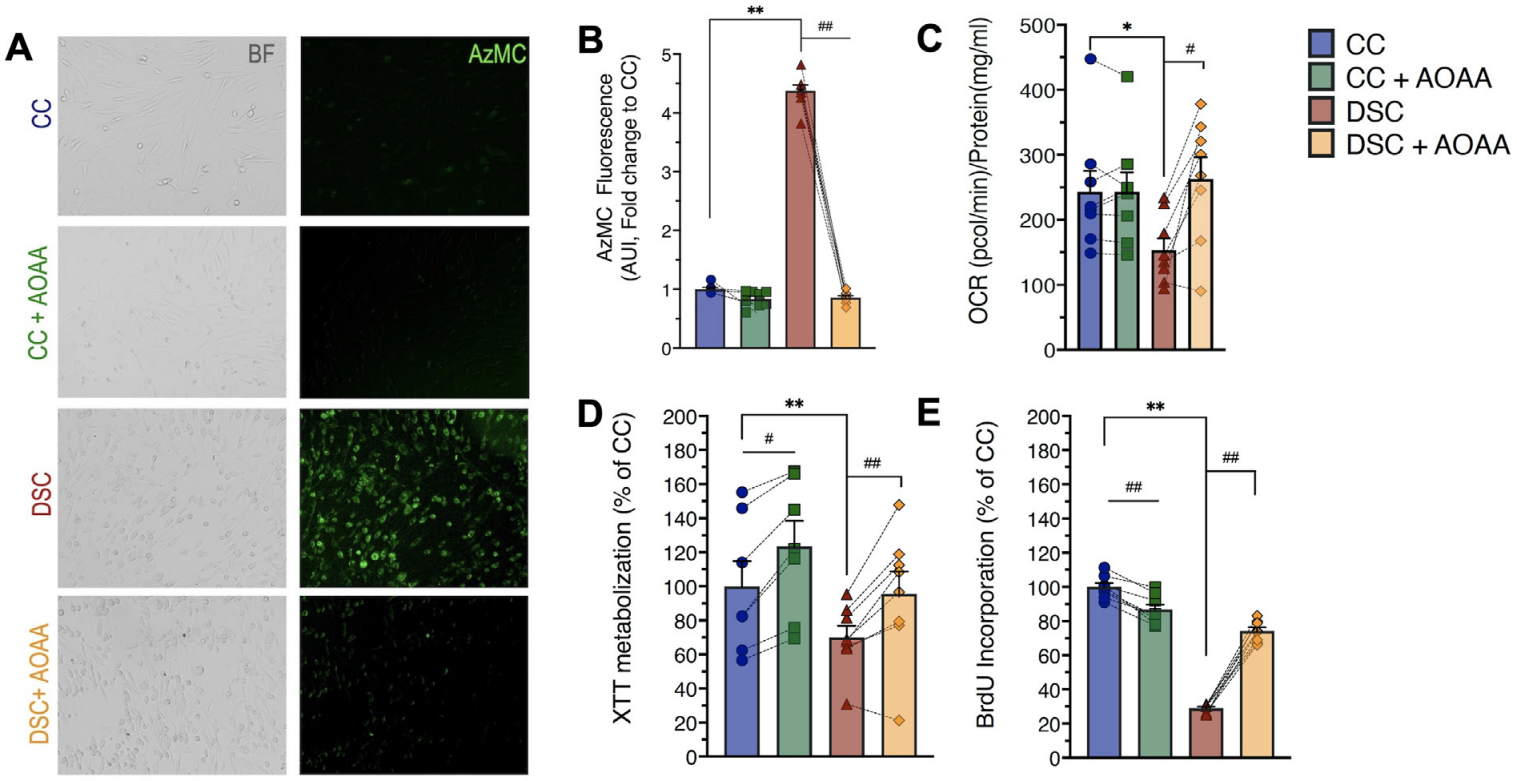
**CBS overexpression in DS is associated increased H_2_S generation in human dermal fibroblasts, which inhibits cellular bioenergetics and suppresses cell proliferation. (A)**: Representative brightfield (BF) images and corresponding images of the fluorescent signal of the H_2_S-specific probe, AzMC, along with **(B)**: the quantification of the AzMC signal in human fibroblasts. **(C)**: Rates of oxygen consumption rate (OCR), **(D)**: XTT metabolization, a marker of mitochondrial function and overall cell viability and **(E)**: BrdU incorporation a marker of cell proliferation in human fibroblasts. The bar graphs represent n ​= ​8 human euploid control fibroblasts (CC) and n ​= ​8 human DS fibroblasts (DSC). Dotted connecting lines indicate the same cell from a specific donor with/without treatment with the CBS inhibitor AOAA (3 ​μM, 24 ​h). ∗∗p ​≤ ​0.01 indicates significant differences between DSC untreated vs. CC untreated; ^#^p ​≤ ​0.05 and ^##^p ​≤ ​0.01 indicate significant differences between CC ​+ ​AOAA vs. CC untreated or DSC ​+ ​AOAA vs. DSC untreated. Reproduced by permission from Ref. [25].

According to the Kamoun Hypothesis (Fig. 1), excess H_2_S, — to a significant part, due to the inhibition of mitochondrial Complex IV — contributes to the pathogenesis of DS via a global bioenergetic effect [9,10,20]. In 2019 we have directly tested this hypothesis by measuring mitochondrial oxygen consumption and ATP production — as well as specific mitochondrial Complex IV activity — in a human DS fibroblast line, as compared to a healthy control fibroblast line. The results demonstrated a marked suppression of mitochondrial respiration in DS cells and revealed that pharmacological inhibition of CBS or its siRNA-mediated silencing improves mitochondrial Complex IV activity, cellular oxygen consumption and ATP generation (Fig. 11) [18]. Subsequent expansion of the study to 8 DS lines vs. 8 control lines [25] demonstrated that **(a)** all of the investigated DS lines exhibit increased H_2_S and polysulfide generation; **(b)** all of the DS lines studied exhibit suppressed mitochondrial electron transport and Complex IV activity; **(c)** pharmacological inhibition of CBS reduces H_2_S generation in these cells and, concomitantly; **(d)** improves mitochondrial electron transport via restoration of specific mitochondrial Complex IV activity (Fig. 12) [18,25]. In contrast, in control cells CBS inhibition or CBS silencing only exerts minor functional effects [18,25]. Importantly, addition of high concentrations of a pharmacological H_2_S donor compound mimics the DS phenotype: the effects include mitochondrial Complex IV, suppression of electron transport, inhibition of aerobic ATP generation and inhibition of cell proliferation [18,25]. Additional effects of CBS inhibition in DS cells included a reduction in mitochondrially-generated oxidative stress [25] — most likely due to the fact that after the 'cloud' of H_2_S is 'lifted' off the mitochondria, electron flow resumes and electrons no longer 'back up' in the mitochondrial electron chain and form various free-radical-generating 'side reactions'. The findings of the above metabolic and fluxomic analysis in DS cells are in partial agreement with an independent study of Anderson and colleagues [101] — conducted predominantly in fibroblasts obtained from DS newborns and from young DS individuals.

**Fig. 13:**
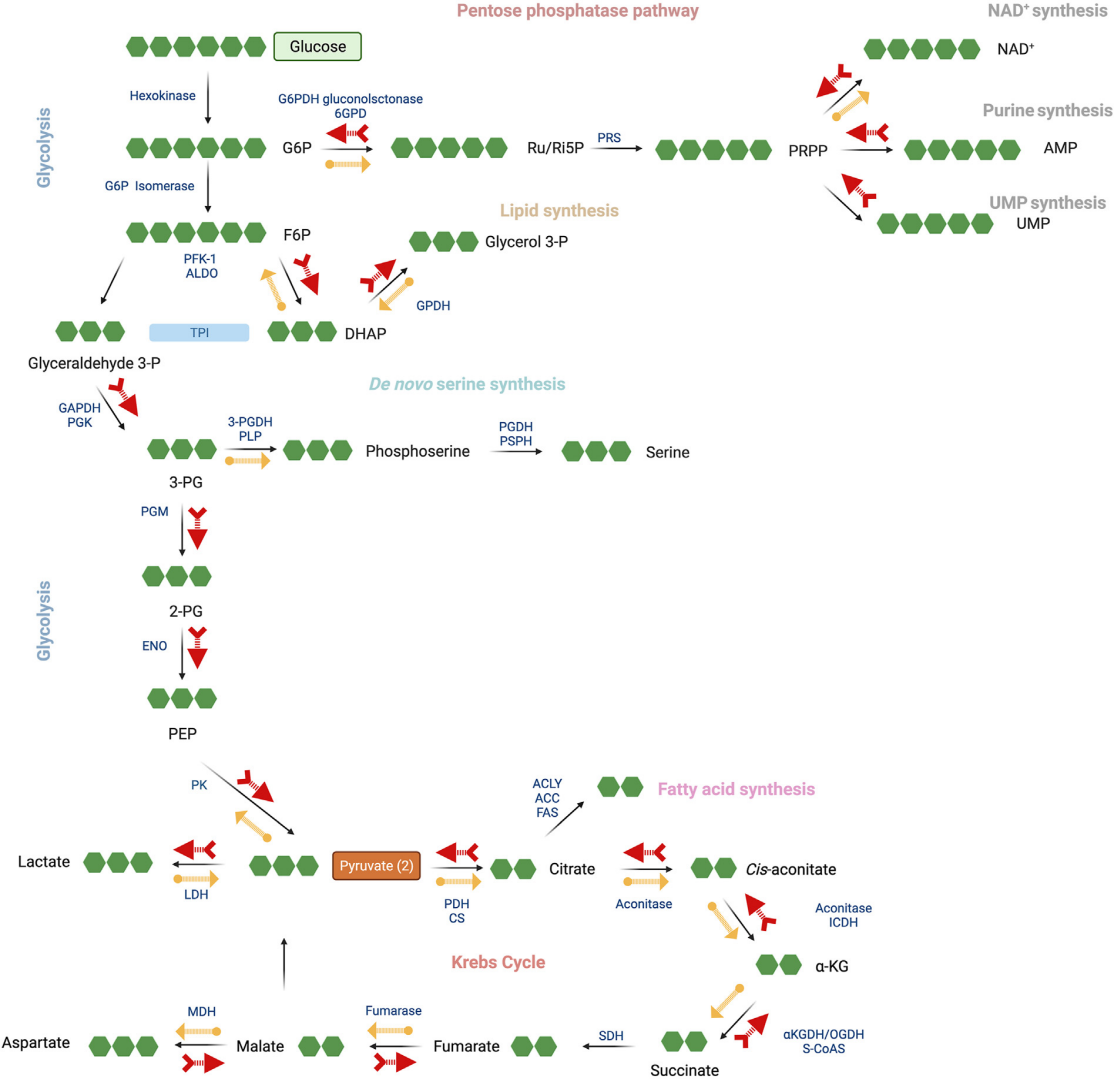
**Summary of the DS-associated central carbon metabolism and related pathways assessed by the fluxomics method.** Blue arrows: normal direction of the fluxes under physiological conditions. Red arrows: shifts induced in DS; yellow arrows: shifts in DS cells induced by AOAA. Data represent the summary of the results of a fluxomics analysis performed in n ​= ​8 human euploid control fibroblasts and n ​= ​8 human DS fibroblasts with/without treatment with the CBS inhibitor AOAA (3 ​μM, 24 ​h). Reproduced by permission from Ref. [25].

Fluxomics analysis [25] also identified significant differences in numerous intracellular metabolites in DS, indicating that CBS upregulation and H_2_S overactivation has wide-ranging consequences in this condition (Fig. 13). Many of the observed metabolomic alterations are consistent with the H_2_S-mediated suppression of mitochondrial electron transport discussed above. DS cells show a significant suppression of ^13^C fluxes into the Krebs cycle, compared to healthy control cells. At the same time, there are marked increases in carbon fluxes into glycolysis metabolites, likely representing a compensatory shift; this shift is also evidenced by increased lactate output (as well as increases in fluxes into glycerol-3-phosphate, dihydroxyacetone phosphate, 3-phosphoglycerate, 2-phosphoglycerate, phosphoenolpyruvate and pyruvate). In contrast with the enhanced glycolytic fluxes, Krebs cycle metabolites — citrate, *cis*-aconitate, alpha-ketoglutarate, malate — exhibit reduced fluxes in DS cells (Fig. 13), consistently with the suppressed mitochondrial electron transport chain activity in DS, which decreases the requirement of the mitochondria for electron donors. Importantly, pharmacological inhibition of CBS reverses most of these effects; this is consistent with the reversible model of Complex IV inhibition by CBS. These findings suggest that – once the "H_2_S cloud" is "lifted off" from the mitochondrial Complex IV, cellular metabolism can reconstitute and aerobic ATP generation can resume.

It is well known that glycolysis is a markedly less efficient way to generate ATP than oxidative phosphorylation that oxidative phosphorylation (2 molecules vs. 36 molecules of ATP generated from each consumed glucose molecule). It is less known that the *speed* of glycolysis is substantially faster than the speed of oxidative phosphorylation — i.e., in glycolysis, substantially more glucose molecules utilized over a given time [126,127]. Thus — as long as the sufficient amount of glucose can be supplied to a cell and the waste product, lactate does not accumulate to toxic levels — glycolysis can generate significant amounts of ATP, which can sustain essential functions in most cell types. This, however, is *not* the case for neurons because for this cell type, mitochondrial ATP generation is essential: this is extremely relevant for the pathobiology of DS, as discussed later.

The results of the fluxomic study of DS cells also indicate the activation of the pentose-phosphate pathway [25]. This metabolic shift has also been demonstrated in DS neurospheres [107], as evidenced by increased carbon fluxes into ribose/ribulose-5-phosphate. DS cells may generate NADH via this pathway, perhaps in another compensatory action to balance the reduced activity of the Krebs cycle. In fact, in a cancer cell model, forced CBS expression was previously found to induce a metabolic shift toward the pentose-phosphate pathway [123,124]. Importantly, CBS inhibition reversed many of the DS-associated fluxomic shifts, including those into the Krebs cycle and into the proximal (oxidative) phase of the pentose phosphate pathway; it also partially restored the DS-associated suppression of *de novo* NAD ​^+^ ​synthesis (Fig. 13).

Taken together, the multiple lines of *in vitro* evidence discussed above demonstrates that **(a)** administration of exogenous H_2_S to normal control cells can recapitulate the cellular bioenergetic phenotype observed in DS cells and **(b)** CBS inhibition or CBS silencing can restore the suppressed bioenergetic function of DS cells, which then reflects in functional outcomes such as improved cell proliferation. Additional data show that pharmacological inhibition of the other major H_2_S producing enzyme, 3-MST can also improve bioenergetic profile in DS cells [21] — although by a relatively smaller degree compared to the effect of CBS inhibition. Moreover, other interventions aimed at normalizing excess H_2_S levels, for example by overexpression of SOD1, or by treatment of the cells with pleiotropic oxidized carbon nanozymes (which both catalytically oxidize H_2_S and dismutate superoxide radicals) also exert beneficial effects in DS cells due to their ability to reduce the "H_2_S burden" of these cells [29]. These findings are consistent with the hypothesis, that H_2_S overproduction, chiefly from CBS upregulation/activation, and to a lesser degree, from 3-MST, is a significant factor in suppressing the metabolism of DS cells. Since cellular ATP generation is an essential factor in supporting and maintaining various vital functions of mammalian cells, it is obvious that impairments in bioenergetics would lead to impaired cell functions, which, in the brain, would be reflected in delayed and impaired neuronal development, impaired synthesis and release of neurotransmitters, a propensity for accelerated cell death and/or neurodegeneration. One could draw parallels between the CBS/H_2_S-mediated metabolic impairments in DS with the neurodevelopmental and functional effects of chronic H_2_S or cyanide poisoning – based on the fact that both of these gases exert their toxic effects via inhibition of mitochondrial Complex IV [120,128,129]. Indeed, the toxicological literature contains numerous examples of metabolic disturbances, neurological developmental problems, impaired central nervous function in the context of H_2_S or cyanide poisoning [[130], [131], [132], [133], [134], [135], [136], [137], [138], [139], [140], [141], [142], [143], [144]].

Nevertheless, it would be an over-simplification to suggest that DS equals a H_2_S-mediated mitochondrial inhibition for several reasons. First of all, H_2_S exerts multiple biological effects, which go well beyond the inhibition of Complex IV activity, including transcriptional effects, effects related to mitochondrial DNA integrity and to posttranslational modification of proteins via SH group persulfidation [reviewed in 10,14–17,20]. A selection of these effects is listed in Table 2. It is conceivable that some or perhaps many of these effects may also contribute to the altered cell function in DS, although these possibilities remain to be investigated. There are multiple mechanisms by which H_2_S can affect various signal transduction and gene transcription processes [17]: these effects may be responsible for some of the diverse alterations in the expression of multiple proteins in DS [25,27]. Pharmacological inhibition of CBS was found to reverse many seemingly disparate, but certainly broad and likely functionally relevant changes in proteome and metabolome of DS cells and tissues. For example, in DS fibroblasts, CBS inhibition induced a significant enrichment of several classes of proteins, belonging to the following cellular pathways: hippo signaling, RAF−independent MAPK1/3 activation, formation of ATP by chemiosmotic coupling, proteins regulating centrosome and microtubule organization and maturation, mitochondrial and other organelle biogenesis and maintenance, Krebs cycle and mitochondrial electron transport chain [25,27]. We do not yet fully understand how and why H_2_S induces and CBS inhibition restores the above shifts in signal transduction and metabolism.

**Table 2:** **Potential molecular targets and possible biological consequences of excess H_2_S in DS.** The table represents an updated version of a table previously published in Ref. [20], which is reproduced by permission.

Target	Effect	Potential relevance for DS
Cytochrome C oxidase	Reversible, tonic inhibition	Inhibition of electron transport, impairment of aerobic ATP generation, suppression of cellular bioenergetics and impaired cell functions, including effects on ER stress and autophagy. Further potential effects: increased mitochondrial ROS production and mitochondrial DNA damage
Hemoglobin	Sulfhemoglobin formation	Decreased O_2_ carrying ability of red blood cells may diminish O_2_ transport to the tissues
Protein cysteine SH residues	Changes in the SH/SSH balance of protein cysteine groups (predominantly, decreased persulfidation)	Multiple protein functions, and, consequently, multiple cellular functions can be affected: cell signaling, cell death and metabolism. These alterations may adversely affect neurodevelopment, neurotransmission, and many other biological functions
DNA	DNA strand breakage and oxidative DNA base modifications	Mutations, cell cycle arrest, developmental and metabolic defects
Promoters and histones	Modulation of promoter activity and/or histone deacetylase activity	Dysregulation of chromatin remodeling, global changes in gene expression, affecting various cell functions
Na^+^/K^+^ ATPase	Inhibition of enzymatic activity	Cellular bioenergetic dysfunction
Carbonic anhydrase	Inhibition of enzymatic activity	Dysregulation of acid-base balance, disturbed transport of CO_2_ away from tissues during respiration, dysregulation of neuronal lipid biosynthesis
Monoamine oxidase	Inhibition of enzymatic activity	Pathological increases in CNS catecholamine and serotonin levels, neuronal dysfunction
Acetylcholinesterase	Inhibition of enzymatic activity	Increased CNS acetylcholine levels, disturbed synaptic transmission
RAC1	Activation	Disturbances of synaptic vesicle release, inhibition of neurotransmitter release
Membrane potassium and calcium channels	Activation or inhibition	Disturbances in membrane potential, adverse effects on multiple cell functions. Potential neuronal calcium overload and associated neurotoxicity
Synaptosomal l-glutamate transporters	Inhibition	Neuronal dysfunction
NMDA receptors	Activation due to increased CNS glutamate levels	Excitotoxicity, neuronal dysfunction
Nuclear transcription factors	Increased gene transcription, inflammatory mediator production	Inflammatory mediator imbalance, immune system disturbances
TRPA1, Cav 3.2	Activation and induction of pro-nociceptive effects	Increased pain sensitivity
cGMP system	Increased cGMP levels	Adverse effects on various intracellular signaling pathways

There is some recent experimental evidence demonstrating altered protein persulfidation in the brain in a mouse model of DS [27]. To our surprise, however, the predominant pattern was a *decrease,* rather than the expected increase in persulfidated proteins. We have already conducted an untargeted proteomics analysis in DS cells and DS brains and investigated the effect of CBS inhibition (which demonstrated a host of functionally important alterations, see below). However, we have not yet investigated how various posttranslational protein modifications change in DS; it is possible that proteins in DS may also undergo other posttranslational modifications (such as oxidation, nitrosylation, nitration, etc.) and the reduced protein persulfidation observed in DS will be better understood once a complete picture of these alterations is obtained. Nevertheless — since, depending on the affected protein —persulfidation can either increase or decrease the activity and/or stability and/or localization and/or interactions of the protein [145], the reduced protein persulfidation in DS is likely have pluripotent functional effects. In a general sense, protein persulfidation has been identified to act as a general stimulator of protein phase separation, i.e., an inhibitor of protein aggregation — as evidenced by the example of several important CNS proteins. Via such a general effect, lower persulfidation may promote the formation of neurofibrillary tangles [146]. Whether such an action may contribute to DS-associated neurodegeneration remains to be explored.

Endoplasmic reticulum (ER) stress occurs when the endoplasmic reticulum cannot properly fold proteins due to the accumulation of misfolded or unfolded proteins, triggering the unfolded protein response (UPR) to restore normal function or initiate apoptosis if stress persists. It is closely linked to autophagy, a process that degrades damaged organelles and misfolded proteins to alleviate stress, often activated by UPR signaling pathways like PERK-eIF2α-ATF4 [[147], [148], [149], [150], [151]]. Both ER stress and autophagy are highly dependent on cellular bioenergetics, particularly mitochondrial ATP generation, as the ER requires ATP for protein folding and chaperone function, while autophagy demands ATP for autophagosome formation, vesicle trafficking, and lysosomal fusion. Mitochondrial dysfunction exacerbates ER stress by impairing ATP supply, and ER stress can disrupt mitochondrial function through disturbed calcium signaling, creating a vicious cycle [[147], [148], [149], [150], [151]]. The above processes have been closely linked with the H_2_S pathway in various cells and tissues, including neurons [[152], [153], [154], [155], [156]]. Thus, it is not surprising that there are important connections of the CBS/H_2_S system in DS with the process of autophagy and ER stress — as demonstrated both *in vitro* in DS fibroblasts [25] and *ex vivo* in a murine DS model [27]. The major changes in the above pathway that are restored by pharmacological CBS inhibition in the fibroblast model are **(a)** restoration of the DS-associated suppression of the expression of ATG7; **(b)** restoration of the DS-associated suppression of beclin-1 phosphorylation on Ser 15 and **(c)** restoration of the restoration of the DS-associated suppression of the expression of lipidated, autophagosome-associated LC3-II [27], while in the *in vivo* model (DS mice) the effects of pharmacological inhibition of CBS include **(d)** restoration of the DS-associated increase of PERK phosphorylation on Thr280; **(e)** restoration of the DS-associated suppression of the expression of BiP and **(f)** prevention of the DS-associated increase of ERO1-Lα expression [27]. Thus, pharmacological inhibition of CBS in DS restores critical disruptions in autophagy and ER stress pathways. It rescues autophagy by restoring ATG7 expression, crucial for autophagosome formation; it suppresses beclin-1 phosphorylation, which may reactivate autophagy initiation; and it affects LC3-II levels, predicting improved autophagosome formation and turnover. Concurrently, CBS inhibition mitigates ER stress by enhancing PERK phosphorylation, a key adaptive response to ER stress; restores BiP expression, which supports protein folding and reduces stress; and suppresses ERO1-Lα, thereby potentially reducing oxidative stress burden. Together, CBS inhibition would be expected to improve cellular homeostasis, reduce the risk of neurodegeneration and oxidative damage, and potentially enhance neuronal functions in DS.

While the Kamoun hypothesis — i.e. a H_2_S-mediated suppression of oxidative phosphorylation and a compensatory increase in glycolysis (Fig. 1) — may explain many pathophysiological alterations in DS, in the CNS, as well as in the periphery (including, quite possibly, the well-known reduction in exercise capacity of DS individuals) it should also be kept in mind that different cell types rely differently on oxidative phosphorylation vs. glycolysis. In the brain, neurons are highly dependent on oxidative phosphorylation, while glial cells predominantly utilize glycolysis [[157], [158], [159], [160]]. Thus, it is a conceivable hypothesis that the excess H_2_S produced in the glia does *not* impair glial cell metabolism, or perhaps – based on *in vitro* effects of H_2_S in glial cells [161] – may even *stimulate* their proliferation. On the other hand, H_2_S that diffuses out of the glial cells to reach the neurons may exert neurotoxic effects via inhibition of mitochondrial ATP generation, disrupting their differentiation and function. This working hypothesis — which does not take into account some further complexities, e.g., that the lactate released by the glia may be taken up by neurons and may in part, help compensate for a H_2_S-mediated suppression of oxidative phosphorylation — remains to be experimentally tested. The above working hypothesis could explain the seemingly paradoxical *in vivo* findings, observed both in the rat and the mouse models [25,27], demonstrating that treatment with a CBS inhibitor reduces DS-associated reactive gliosis.

Obviously, H_2_S overproduction and its downstream effects are not the sole relevant metabolic alterations in DS. As previously discussed, DS is characterized by a global reprogramming of cellular genomes, involving the upregulation and downregulation of thousands of genes across all chromosomes — not limited to those encoded by chromosome 21 (Fig. 17). We propose that the alterations driven by H_2_S overproduction occur against a backdrop of widespread cellular adaptations and maladaptations. These processes likely include various mitochondrial disturbances (e.g., the dysregulation of various mitochondrial proteins, and alterations in mitochondrial transporters and metabolites), which may predispose or sensitize mitochondria to H_2_S-induced metabolic impairments. Nevertheless, experimental evidence from various *in vitro* (see above) and *in vivo* (see below) models suggests that pharmacological inhibition of CBS can, in itself, significantly improve multiple metabolic and functional outcomes. This improvement occurs despite the presence of other maladaptive changes, indicating that CBS inhibition may be able to "tip the balance" of key metabolic processes, and thereby improving cellular metabolic homeostasis.

**Fig. 14:**
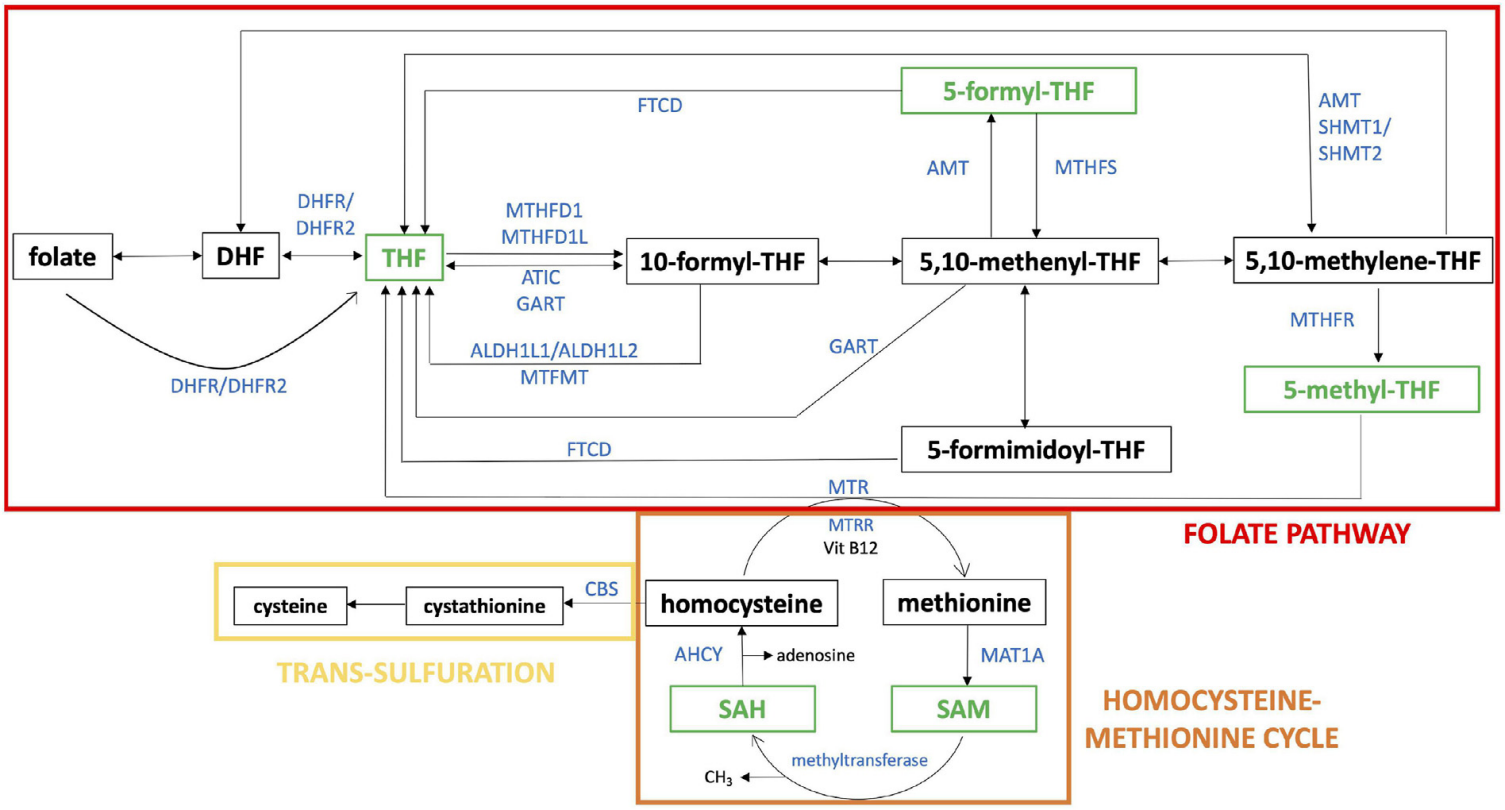
**The relationship of CBS with various components of the one-carbon pathway**. The schematic representation of folate pathway (in red), homocysteine-methionine cycle (in orange) and transsulfuration pathway (in yellow) are shown. Reproduced by permission from Ref. [71].

It should be emphasized that CBS, or, in a broader sense, the reverse transsulfuration pathway, is closely integrated with essential components of the one-carbon pathway, such as the folate pathway and the homocysteine-methionine cycle (Fig. 14). In addition, downstream from CBS, the reverse transsulfuration pathway is closely linked to metabolism of glutathione, taurine and many other metabolites that regulate essential processes in mammalian cells. Thus, overexpression of CBS in DS has substantially broader functional roles than generating H_2_S at a higher rate, and inhibition of CBS also has substantially broader effects than reducing the cellular levels of this mediator. Some of these effects may be directly related to changes in cellular homocysteine levels; others — including changes in gene expression — may be mediated, in part, by decrease epigenetic methylation processes via influencing the balance between S-adenosylmethionine (SAM) and S-adenosylhomocysteine (SAH), two metabolites critical for epigenetic regulation and/or via gene expression effects associated with the folic acid-dependent homocysteine re-methylation process [30,71]. It is therefore not surprising that overexpression of CBS exerts broad effects on various cellular metabolite levels, including glutathione metabolism and cellular redox balance, as well as pluripotent effects on gene expression [23,50,162,163]. For example, in one study, CBS overexpression in the NCM356 colonic epithelial cell line induced broad changes in the transcriptome, with over 350 differentially expressed genes related to glycolysis, hypoxia, and colon cancer cell phenotypes. In the same study, metabolomic analysis revealed alterations in metabolites associated with glycolysis, nucleotide sugars, the pentose phosphate pathway, and lipogenesis, including phospholipids, sphingolipids, and bile acids [162]. In DS, the various CBS-associated metabolic and gene expression processes should *not* be considered in isolation, but, rather, should be viewed on the background of the complex reprogramming of the cell's transcriptional and metabolic processes discussed in the previous paragraph. These effects must also be considered when considering the role of CBS in DS and the functional effect of CBS inhibition in this condition.

## Functional role of H_2_S in DS: *in vivo* and *ex vivo* studies

The genes encoded on human chromosome 21 are distributed across three rodent chromosomes — chromosomes 10, 16, and 17 — representing approximately 80 ​%, 15 ​%, and 5 ​% of these genes, respectively. The gene encoding CBS, which is located in the subtelomeric region of human chromosome 21, is found on the proximal region of mouse chromosome 17 [164]. Historically, many mouse models of DS are not directly relevant to CBS or H_2_S biology because they lack triplication of the chromosome segment encoding CBS. For instance, the Ts65Dn mouse model, which was created by translocating a segment of mouse chromosome 16 containing approximately 100 homologous human chromosome 21 genes, remains one of the most widely used DS models, with over 500 published studies [165,166]. Although Ts65Dn mice do not carry three copies of CBS, they exhibit numerous hallmark DS features, including cognitive deficits, learning and memory impairments, abnormalities in synaptic plasticity, craniofacial dysmorphologies, motor coordination deficits, and early-onset neurodegeneration. They also display cardiac defects, immune dysregulation, and altered neurogenesis.

The absence of CBS triplication in the Ts65Dn model does not invalidate the CBS/H_2_S pathogenetic concept in DS. Instead, it highlights that diverse gene triplication patterns can disrupt development and lead to DS-like functional impairments. Ts65Dn mice exhibit trisomy for several other critical DS-related genes, such as DYRK1A, SOD1, and APP, which independently contribute to overlapping or complementary pathological features. These factors may induce significant developmental and functional defects, even in the absence of CBS triplication in this model. It should also be mentioned that approximately 60–70 non-syntenic genes — located in regions of mouse chromosome 16 outside the syntenic region corresponding to human chromosome 21 — are also triplicated in the Ts65Dn model, potentially influencing its phenotype.

Similar limitations exist in other mouse models of DS. Many models feature only partial triplication of human-equivalent genes, while others include triplication of genes absent from human chromosome 21. Attempts to triplicate all human chromosome 21 genes in mice have resulted in models so fragile that experimental studies are often impractical. Introducing a full human chromosome 21 into rodents generates additional gene products, but introduces other challenges, such as immune incompatibility and mosaicism. For an in-depth overview of various DS rodent models, the reader is referred to specialized reviews [[44], [45], [46], [47],^165^,^166^]. In the following sections, we will focus on mouse models of DS that include triplication of the chromosome segment encoding CBS.

CBS is prominently expressed in the brain and plays a critical role in regulating gene expression and metabolism in a cell-type- and region-specific manner, contributing to neurodevelopment and CNS function [4,[167], [168], [169], [170], [171], [172], [173], [174], [175], [176], [177], [178], [179], [180], [181], [182], [183], [184], [185], [186], [187]]. Although CBS expression has been demonstrated in various brain cell types, including neurons and cerebral vasculature cells, its highest expression is observed in the glia [13,24,27,78,174]. Despite this, global CBS deletion in mice (Cbs^tm1Unc/+^ knockout model) does not appear to significantly impact CNS function. These animals exhibit normal locomotor learning in the rotarod test and retain their ability to recognize novel objects, comparable to wild-type mice [19].

One approach to explore CBS's functional role in DS pathophysiology is to overexpress the enzyme, either globally or in a cell-specific manner. Early studies employing transgenic mice carrying human CBS cDNA under the control of a metallothionein promoter found no significant disturbances in sulfur amino acid metabolism, motor coordination, balance, or motor learning ability. Notably, hippocampal synaptic plasticity — measured through long-term potentiation — was enhanced [188,189]. However, it should be noted that the use of a metallothionein promoter to regulate CBS expression makes this model somewhat artificial, as it does not account for endogenous regulatory mechanisms such as promoter activity or alternative splicing.

A subsequent transgenic mouse model expressed the human CBS gene under murine endogenous regulatory control, specifically to investigate DS pathogenesis [190]. However, studies on neurodevelopment or CNS function in this model have not been reported to date. Another study using hCBS transgenic mice associated CBS overexpression in various brain regions reported significant alterations in serotonin and dopamine pathway metabolites, but this study did not investigate neurodevelopment, memory, or behavior [191].

In 2019, Herault and colleagues demonstrated that CBS overexpression alone is sufficient to induce deficits in novel object recognition tests in mice, without affecting locomotor activity in the rotarod test [19]. The CBS-overexpressor mice also exhibited increased circadian activity compared to wild-type controls. In this model, CBS overexpression was specifically targeted to Camk2a-expressing excitatory glutamatergic neurons, which are predominantly in the hippocampus and cortex (but not in the cerebellum). Thus, neuron-specific CBS overexpression is sufficient to impair object memory, supporting the potential role of CBS in DS. Neurodevelopmental abnormalities or gross morphological differences were not reported in this mouse strain. While the findings obtained with the above model are valuable, it should be re-emphasized that both in healthy mammals and in DS, CBS (over)expression occurs primarily in glial cells, i.e., *not* in neurons. Nevertheless, as H_2_S is a diffusible signaling molecule, glia-derived H_2_S can impact neighboring neurons, lending some validity to this model for studying DS. Future studies focusing on astrocyte-selective CBS overexpression in mice would provide further insights into CBS's role in DS pathophysiology.

An alternative approach to studying CBS in DS involves mouse models of trisomy that include triplication of mouse chromosome 17, which encodes CBS. The first study investigating chromosome 17 genes in DS used the Ts43 ​H ​Ph mouse segmental trisomy model, published in 2005 [192]. This model included triplication of 310 known genes in the proximal 30 Mbp of mouse chromosome 17. However, the expression levels of genes near the translocation breakpoint, including cbs, nudt3 and fkbp5, did *not* differ from controls. Consequently, while in this model the mice exhibit impaired CNS function — evidenced by severely reduced place navigation learning in the Morris water maze — the model offers limited insights into CBS pathobiology in DS.

The Dp(17Abcg1-cbs)1Yah mouse, also known as Dp1Yah or Ts1Yah, was developed by generating trisomy of 12 genes located in the 0.59 ​Mb Abcg1-U2af1 region of the Hsa21 subtelomeric region, including the CBS gene [193]. In this model, CBS overexpression is functionally reflected by reduced plasma homocysteine levels. Behaviorally, these mice exhibit defects in open-field, Y-maze, and novel object recognition tests but, unexpectedly, show *enhanced* spatial reference memory in the Morris water maze test, coupled with *stronger* hippocampal long-term potentiation (LTP) [193]. These latter findings echo the intensified LTP responses observed in CBS transgenic mice [188], suggesting overlapping mechanistic pathways.

**Fig. 15:**
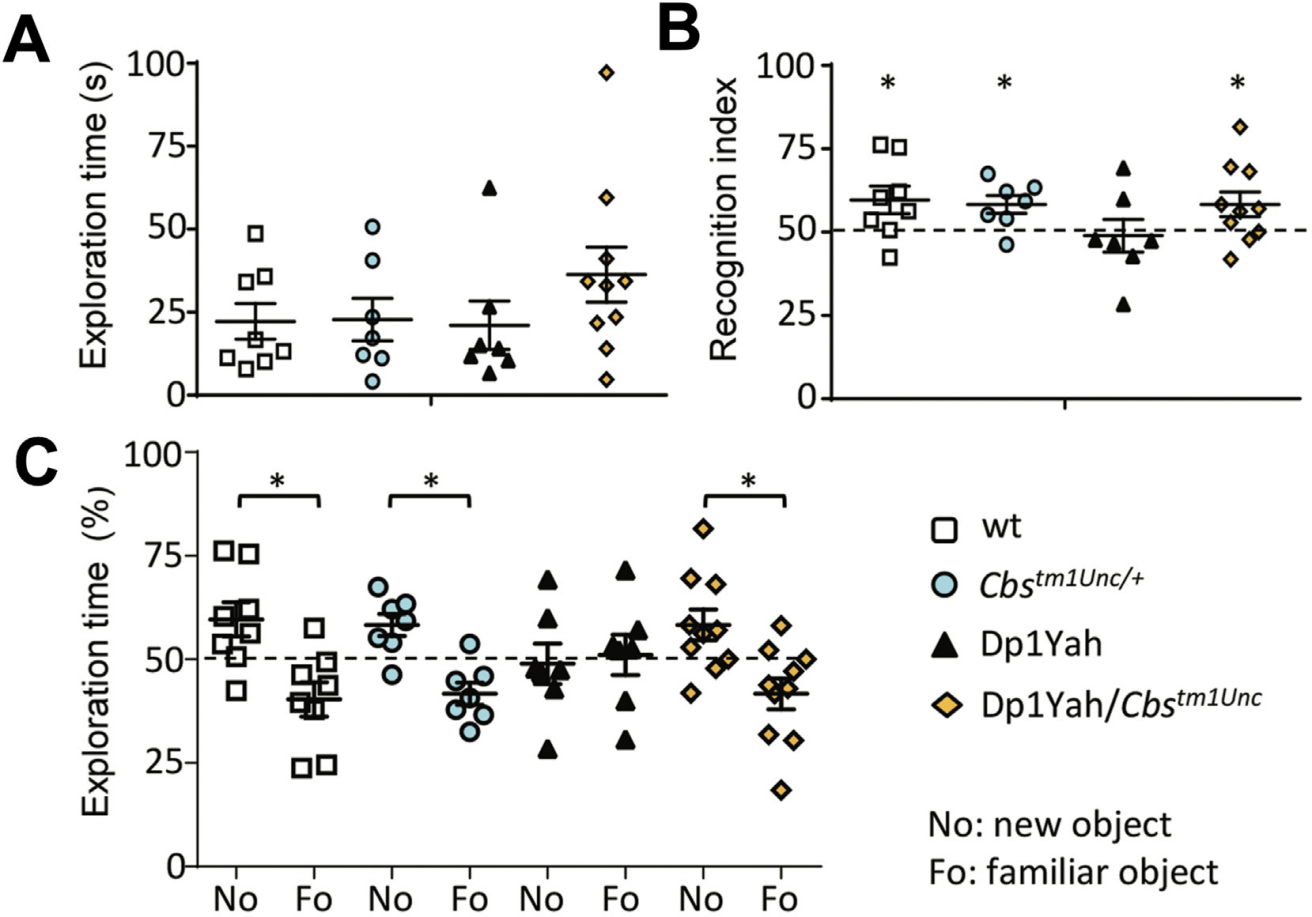
**CBS contributes to the neurocognitive dysfunction in the Dp1Yah model of DS mouse. Dp1Yah trisomic mice (n ​= ​23) were compared with Dp1Yah carrying a knock-out of Cbs (Dp1Yah/Cbs^tm1Unc^), Cbst^m1Unc/+^ and wild-type controls. (A)**: The exploration time in the first session of the novel object recognition test was not statistically different in the four genotypes but **(B)**: during the recognition phase, after 10 ​min of retention, the recognition index (right upper panel, time spent on the No/total time of exploration) was clearly lower in Dp1Yah mice as compared with the other genotypes and not statistically different from chance (50 ​%). Accordingly, **(C)**: the exploration time spent by the Dp1Yah/Cbstm1Unc mice to explore the object showed that they were able to differentiate the novel (No) versus familiar (Fo) object, while the Dp1Yah were not. ∗p ​≤ ​0.05. Reproduced by permission from Ref. [19].

Subsequent studies further investigated the role of CBS in DS, using the Dp1Yah mice. These mice demonstrate decreased locomotor learning in the rotarod test and impaired novel object recognition [19]. When Dp1Yah mice were crossed with the Cbs^tm1Unc/+^ knockout model [194] to normalize CBS overexpression, the resulting Dp1Yah/Cbs mice exhibited increased distance traveled and higher speeds in open-field and Y-maze tests. Importantly, CBS normalization improved locomotor learning and restored the DS animals' learning and memory function, as shown in the novel object recognition test (Fig. 15) [19]. These results strongly implicate CBS in the neurocognitive deficits associated with DS. Proteomic analysis of Dp1Yah brains revealed upregulation of multiple proteins associated with oxidative stress responses, including SOD1, glutathione S-transferases mu 1 and 5, and peroxiredoxins 5 and 6. Additionally, the expression of glyoxalase I, a key enzyme involved in detoxifying methylglyoxal — a glycolytic byproduct — was markedly upregulated [19]. Methylglyoxal accumulation can lead to protein, DNA, and lipid damage through advanced glycation end-products [195,196]. Thus, glyoxalase I upregulation may represent a protective mechanism against the increased glycolytic flux that occurs in DS cells.

Further metabolic adaptations were observed in this mouse DS model, including upregulation of phosphofructokinase (muscle type), lactate dehydrogenase A and B, cytochrome *c* oxidase subunit VIb, and ATP synthase subunit B1 [19]. Phosphofructokinase, a rate-limiting glycolytic enzyme, catalyzes the conversion of fructose-6-phosphate to fructose-1,6-bisphosphate, while glyceraldehyde-3-phosphate dehydrogenase drives the conversion of glyceraldehyde-3-phosphate to 1,3-bisphosphoglycerate [19]. These changes collectively indicate a metabolic shift toward anaerobic glycolysis in DS. Similarly, lactate dehydrogenase upregulation reflects increased lactate production, a hallmark of enhanced glycolytic activity. The upregulation of cytochrome *c* and ATP synthase subunits may reflect compensatory mitochondrial responses.

Transketolase, an enzyme in the pentose phosphate pathway, was also upregulated in DS mouse brains [19]. By facilitating carbon group transfer between sugars, transketolase supports biosynthetic demands arising from increased glycolytic flux. Additionally, pyruvate dehydrogenase (beta subunit) expression was elevated [19]. While this enzyme typically converts pyruvate into acetyl-CoA for the Krebs cycle, acetyl-CoA may be diverted into alternative pathways, such as fatty acid synthesis or acetylation reactions, under conditions of mitochondrial dysfunction [[197], [198], [199]]. The role of these alternative metabolic pathways in DS require further investigation.

**Fig. 16:**
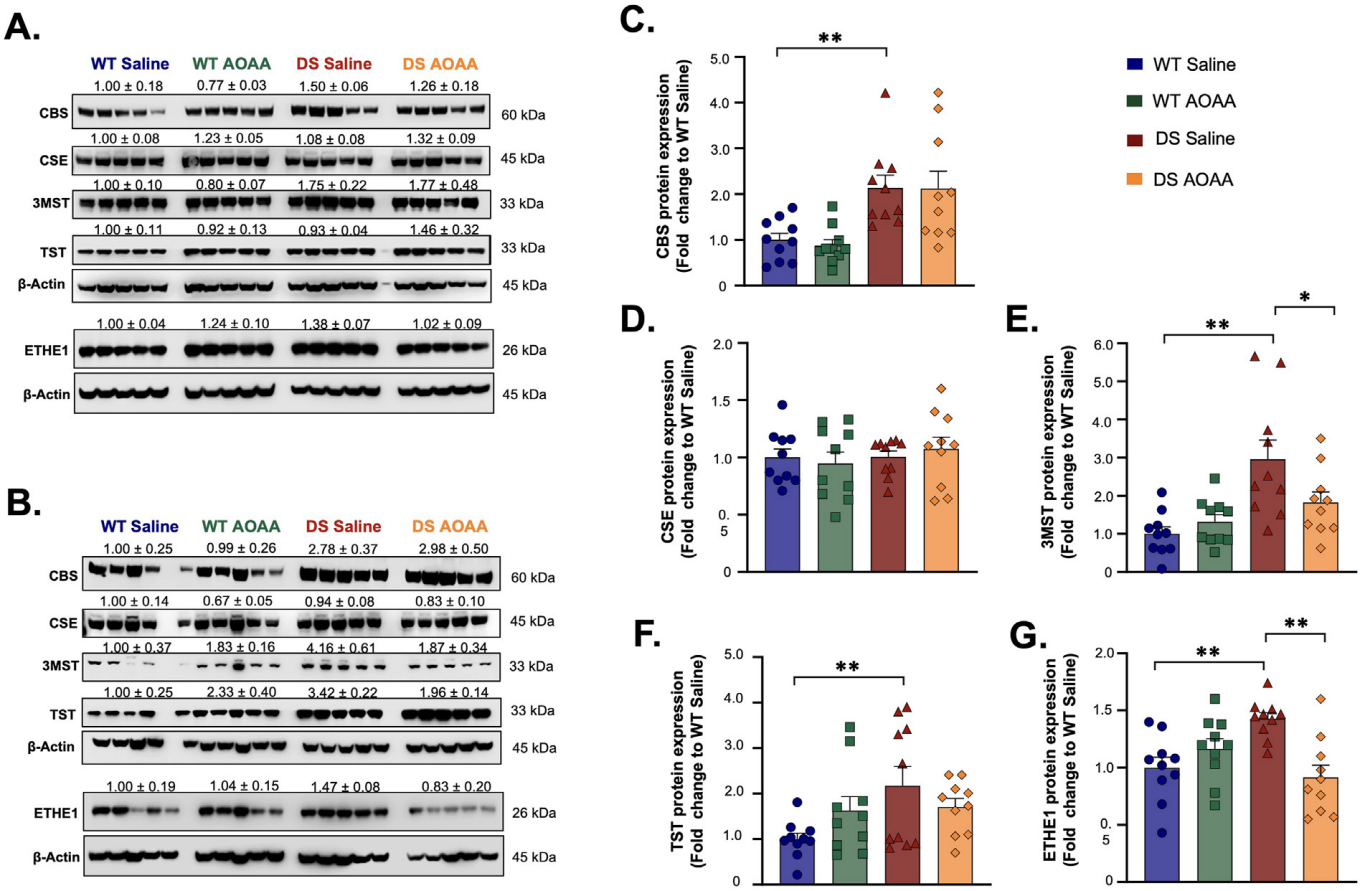
**The brain of DS mice contains higher levels of CBS, 3-MST, TST and ETHE1 than the brains of wild-type control mice. (A**–**B)**: Representative immunoblots of the expression levels of the enzymes for H_2_S metabolism in whole brain homogenates in **(A)** male and **(B)** female wild-type and Dp(17)3Yey/+ DS mice treated with vehicle or the CBS inhibitor AOAA. **(C**–**G)**: Densitometric analysis of the expression of the various enzymes in wild-type or DS mice treated either with vehicle or AOAA. ∗p ​≤ ​0.05, ∗∗p ​≤ ​0.05. Reproduced by permission from Ref. [27].

**Fig. 17:**
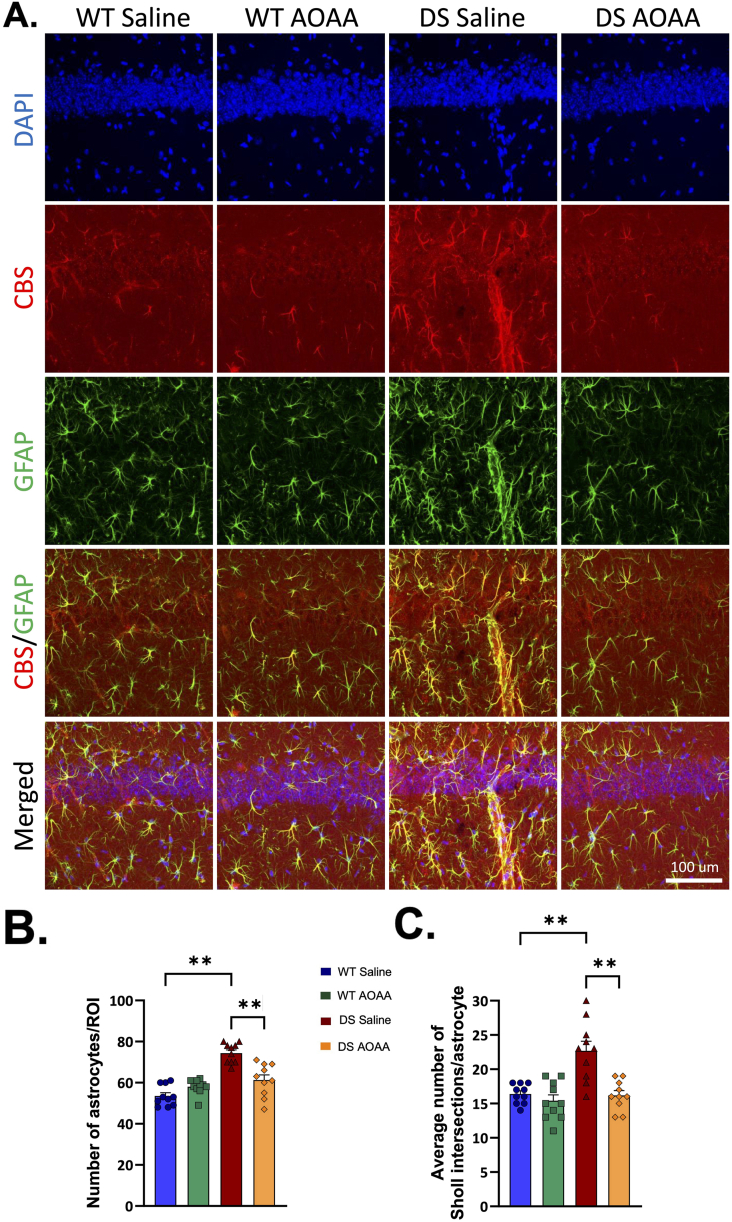
**Reactive astrogliosis in the DS brain**. Immunohistochemical staining of mouse brain sections of wild-type and DS mice (with or without AOAA treatment) was analyzed in the CA1 region of the hippocampus. (A): Representative images; **(B)**: astrocyte density and **(C)**: Sholl analysis of astrocyte intersections. DS was associated with reactive astrogliosis, as evidenced by higher astrocyte numbers, larger astrocyte cell size, and more complex branching. ∗∗p ​≤ ​0.01. Reproduced by permission from Ref. [27].

**Fig. 18:**
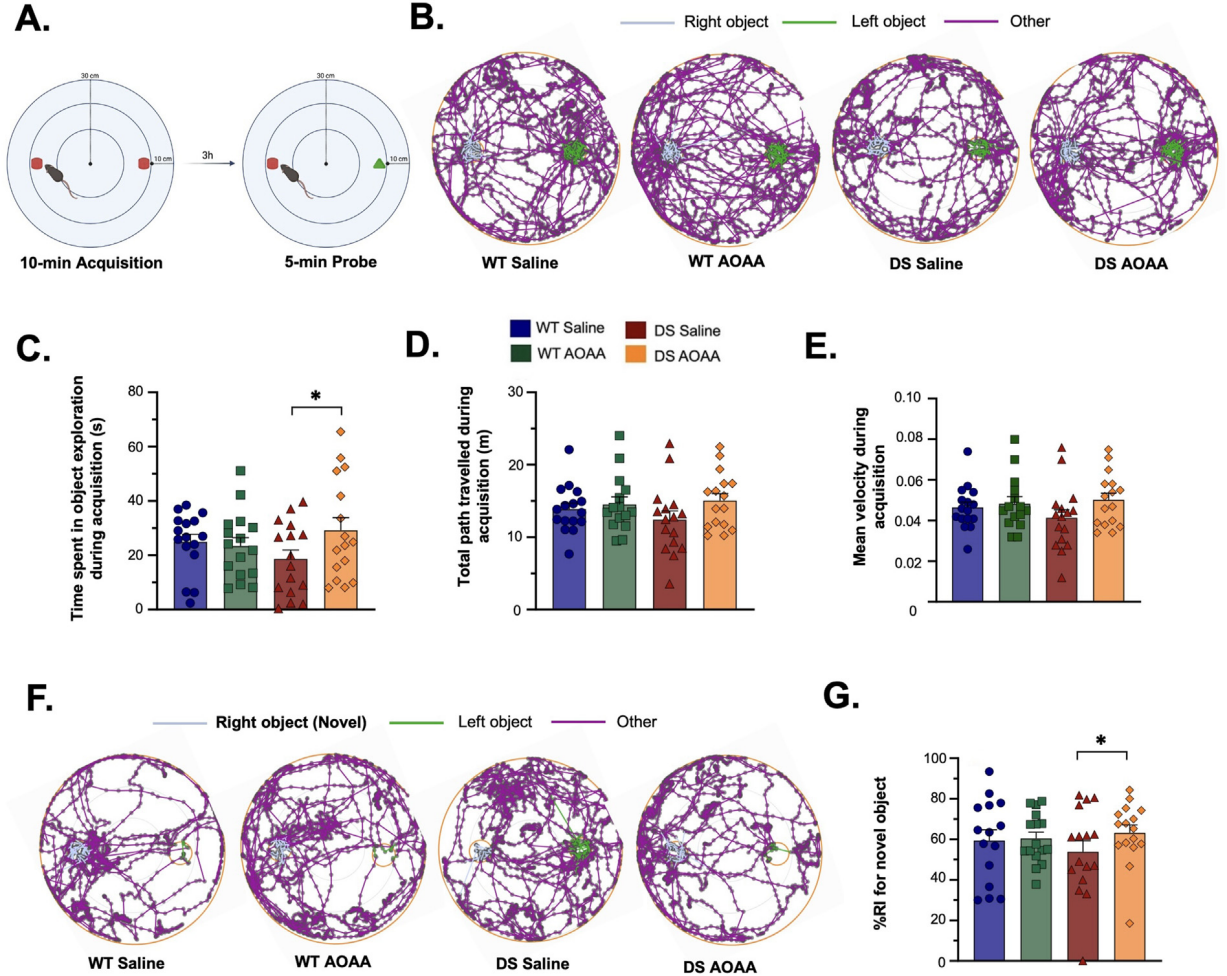
**CBS inhibition improves recognition memory in the Dp(17)3Yey/+ mouse model of DS. (A)**: Illustration of the novel object paradigm used. **(B)**: Representative tracking maps of animal activity in the arena during the 10-min acquisition session. **(C)**: The total time spent in object exploration along with **(D)**: the total path traveled and **(E)**: the mean velocity were recorded during the acquisition trial. **(F)**: Representative tracking maps of animal activity in the arena during the 5-min retention trial. **(G)**: The recognition index (%RI) for the novel object – calculated as a percentage ratio of the time allotted in the novel object over the total object indicates recognition memory. Treatment of the mice with the CBS inhibitor AOAA improved recognition memory. ∗p ​≤ ​0.05. Reproduced by permission from Ref. [27].

In 2010, Eugene Yu's group introduced three mouse models harboring duplications spanning the entire human chromosome 21 syntenic region on mouse chromosomes 10, 16, or 17, respectively [200]. Among these, the Dp(17Abcg1-Rrp1b)3Yey mouse (also known as Dp(17)3Yey, Dp(17)1Yey, or Ts3Yey) includes the triplicated *cbs* gene. This model exhibits enhanced LTP responses, consistent with findings in other models with CBS triplication. Dp(17)3Yey/+ mice display normal locomotion, exploratory behavior, and responsiveness in the Morris water maze and contextual fear conditioning tests. However, they show impairments in recognition memory (novel object recognition test) and spatial learning (T-maze test) [27,200]. CBS expression in Dp(17)3Yey/+ mice is approximately twice as high in the brain compared to wild-type controls — with a more pronounced upregulation in female mice than in male mice (Fig. 16) — and is mainly localized to glial cells (Fig. 17) [27]. Additionally, 3-MST, another H_2_S-producing enzyme, is upregulated alongside the H_2_S-catabolizing enzymes TST (rhodanese) and ETHE1 [27]. Pharmacological inhibition of CBS with aminooxyacetic acid (AOAA) in this model improves recognition memory and spatial learning deficits (Fig. 18) [27]. These improvements are linked to restored mitochondrial function, including normalization of Complex IV activity and expression in synaptic regions, enhanced ATP production, synaptic vesicle recycling, and increased expression of synaptic markers [27]. AOAA also attenuated dysregulated unfolded protein response pathways and reduced activation of autophagy-associated pathways.

Remarkably, even with a short two-week treatment period, AOAA normalized the glial-to-neuronal cell ratio, reducing glial mass and complexity (Fig. 17) [27]. This finding aligns with clinical observations in DS, which consistently report a higher glial cell count and reduced neuronal population [[201], [202], [203], [204], [205], [206]]. Reactive astrogliosis, a key factor in DS and other CNS diseases (e.g., Alzheimer's disease), has been implicated in synaptic dysfunction and neuronal death. Targeting this process pharmacologically is emerging as a potential therapeutic strategy [205,206]. The inhibitory effect of the CBS inhibitor AOAA on gliosis suggest that CBS-derived H_2_S significantly contributes to astrogliosis and the associated mitochondrial dysfunction in DS neurons.

Proteomic analysis of Dp(17)3Yey/+ brains confirmed CBS upregulation and identified elevated levels of glyoxalase I, farnesyl pyrophosphate synthase 1, and ferritin heavy chain [27]. The markedly increased expression of glyoxalase I is likely an adaptive response to elevated glycolytic flux, a finding consistent with observations in the Dp1Yah model discussed earlier. Additional proteomic changes included upregulation of farnesyl pyrophosphate synthase 1 and ferritin heavy chain [27], which may reflect cellular adaptations to oxidative stress and metabolic imbalances. Farnesyl pyrophosphate synthase 1 upregulation suggests alterations in the mevalonate pathway, potentially linking cholesterol and lipid biosynthesis to DS pathology. Similarly, ferritin heavy chain upregulation may indicate enhanced iron storage as a protective mechanism against oxidative stress. In contrast, several critical mitochondrial proteins were downregulated, including cytochrome *c* oxidase subunit 5A and NADH dehydrogenase [ubiquinone] 1 alpha subcomplex subunit 5, highlighting impaired electron transport chain activity [27]. Such disruptions likely contribute to reduced ATP production and increased reactive oxygen species (ROS) generation. Downregulation of dihydrolipoyllysine-residue acetyltransferase, a core component of the pyruvate dehydrogenase complex in DS [27], may impede the conversion of pyruvate to acetyl-CoA, limiting TCA cycle entry and suppressing aerobic ATP generation. Reduced expression of electron transfer flavoprotein-ubiquinone oxidoreductase, a key player in fatty acid and amino acid metabolism in DS [27], suggests diminished mitochondrial β-oxidation capacity, further exacerbating energy deficits.

Importantly, protein function is regulated not only by absolute expression levels but also by their intracellular localization, redistribution, and post-translational modifications. While these aspects were not comprehensively addressed in the study, an exception was protein persulfidation. The analysis revealed widespread decreases in protein persulfidation; increased persulfidation was a relatively rare event, however [27]. Gene ontology analysis linked the affected proteins to pathways involved in metabolism, carbon flux, neurodegeneration, calcium signaling, long-term potentiation, and Alzheimer's disease pathogenesis [27]. While these pathways have established connections to DS, the functional consequences of altered persulfidation remain unclear, warranting further investigation.

Untargeted metabolomic analysis of Dp(17)3Yey/+ brains corroborated the proteomic findings, revealing significant disruptions in mitochondrial function and a compensatory upregulation of glycolysis. Key TCA cycle intermediates, including citrate, aconitate, fumarate, malate, and succinylcarnitine, were markedly reduced, alongside decreased glucose levels and treatment with AOAA significantly restored these metabolites, normalizing citrate, fumarate, and malate levels, indicative of reactivation of the Krebs cycle [27]. This effect likely stems from AOAA-mediated relief of H_2_S-induced Complex IV inhibition, thereby improving mitochondrial respiration and ATP production. Glycolytic intermediates, including 2- and 3-phosphoglycerate, phosphoenolpyruvate, and pyruvate, were also elevated in DS brains [27], reflecting enhanced glycolytic flux. Concurrently, increased levels of 6-phosphogluconate in the DS brain pointed to upregulated *oxidative* PPP activity, likely as a defense against oxidative stress. Conversely, reduced sedoheptulose-7-phosphate and ribose 5-phosphate levels [27] suggested impaired *non-oxidative* PPP activity, potentially limiting nucleotide biosynthesis. AOAA treatment rebalanced PPP activity, as evidenced by reduced 6-phosphogluconate and increased sedoheptulose-7-phosphate levels. A shift toward the PPP as an alternative to glycolysis was similarly observed in human DS fibroblasts in prior fluxomics studies [27].

The metabolomic analysis also revealed significant alterations in amino acid metabolism. Glycine levels were reduced in DS brains, potentially due to decreased biosynthesis or increased consumption, leading to impaired glutathione production and redox imbalances [27]. AOAA treatment restored glycine levels, predicting improved antioxidant capacity [27]. Serine, elevated in DS brains as a compensatory mechanism, was also normalized by AOAA treatment. Histidine metabolism was also altered, with elevated carnosine and reduced homocarnosine levels, indicating disruptions in antioxidant dipeptide metabolism. AOAA treatment enhanced homocarnosine levels, suggesting an additional neuroprotective mechanism [27]. Tyrosine metabolism was similarly perturbed, with elevated dopamine and homovanillate levels contributing to oxidative stress and neurobehavioral abnormalities, both of which were mitigated by AOAA [27]. Polyamine metabolism, crucial for cell proliferation and stress responses, was also impaired, with reduced putrescine, spermidine, and spermine levels. AOAA treatment restored these metabolites, reflecting broader metabolic improvements [27]. Alterations in purine, pyrimidine, and nicotinate metabolism were also observed, with AOAA reducing elevated UDP levels and partially normalizing NAD^+^ and NADH levels [27]. These changes indicate improved redox balance and mitochondrial function after CBS inhibition. The precise mechanisms by which CBS inhibition rebalances these pathways remain unclear but are hypothesized to involve reductions in excess H_2_S, which globally affects cellular bioenergetics and metabolic fluxes.

Lipid metabolism was significantly impaired in DS brains, with reductions in monounsaturated fatty acids (MUFAs), polyunsaturated fatty acids (PUFAs), acylcarnitines, and hydroxylated fatty acids [27]. These alterations predict deficits in lipid synthesis, elongation, and oxidation, potentially impairing neuronal and glial function and repair capacity. Additionally, key endocannabinoid metabolites, such as oleoyl ethanolamide and palmitoyl ethanolamide, were decreased, indicating disrupted lipid signaling pathways. These changes likely contribute to impaired neural communication, regeneration, and stress responses. AOAA treatment normalized many of these lipid metabolites, consistent with its neuroprotective effects [27]. For instance, restored MUFA, PUFA, and acylcarnitine levels reflect improved mitochondrial β-oxidation and energy metabolism. Normalization of endocannabinoid levels suggests enhanced lipid signaling, potentially improving neuronal function and resilience. The link of these effects to H_2_S overproduction or mitochondrial dysfunction remains to be explored. A potential connection may be that AOAA improves mitochondrial function (reflected by restored acylcarnitines), improves the pentose phosphate pathway and/or reduces oxidative stress, all of which could improve various components of the lipid homeostasis.

There are several other DS models as well that include cbs triplication. One model, developed by Eugene Yu, carries duplications spanning the entire Hsa21 syntenic regions on all three mouse chromosomes. This mouse mutant exhibits severe DS-related neurological defects, including impaired cognitive behaviors, reduced hippocampal long-term potentiation and hydrocephalus [207,208]. While there is evidence that mouse chromosome 17 (which, among others, also encodes cbs), plays an important role in the pathogenesis of the various defects in this model [[208], [209], [210], [211]], the specific role of CBS remains to be explored in this model.

Another model, named Ts(1716)65Dn/J (Ts65Dn), displays many phenotypes that appear to mimic human DS features. These animals carry an additional mini-chromosome with the Mir 155 to Zbtb21 region of mouse chromosome 16, homologous to Hsa21, encompassing around 90 genes, fused to the centromeric part of mouse chromosome 17 from Pisd-ps2/Scaf8 to Pde10a. However, this animal model also carries 46 genes *not* related to human chromosome 21 [212]. This latter issue was recently corrected, and an improved version of this model, Ts66Yah, was developed which, using CRISPR/Cas9 deleted the genomic region unrelated to Hsa21 on the mini-chromosome [213,214]. It is hoped that future studies will utilize these models to explore the specific role of various key driver pathways in DS pathogenesis, including the role of the CBS/H_2_S pathway.

There are also several rodent models of DS — some in mice, some in rats — which utilize various human artificial chromosomes, which typically also include the region that encodes for CBS [[215], [216], [217]]. In these models — in concert with a wide range of alterations in gene expression — CBS protein is elevated, and these animals exhibit significant neurocognitive and behavioral defects. However, the specific role of CBS — vs. the multitude of additional triplicated genes — has not yet been directly investigated in these models. The only indication for the potential role of CBS in these models is by Marechal and colleagues from 2015 [218]. In this report, the contribution of the Abcg1-U2af1 genetic interval was investigated on the behavioral phenotypes observed in the Tc1 mouse model of DS. This mouse carries an additional freely segregating human chromosome 21 and exhibits cognitive and locomotor deficits. By generating compound mutants (Tc1/Ms2Yah) with a deletion of the Abcg1-U2af1 interval, some DS-associated deficits were improved, such as improved performance in the Morris water maze and better motor coordination on the rotarod test [218]. The Abcg1-U2af1 region includes 12 genes, one of which is CBS and thus may have contributed to the observed rescue. However, CBS is not the sole gene in this interval, and additional genes may also explain the observed effects. Among these, PDE9A, known for its regulation of cGMP signaling and its critical role in synaptic plasticity, may be a candidate. Similarly, ABCG1, involved in lipid metabolism, could influence neural health and glial function in DS. Other genes, such as RUNX1, associated with immune regulation, and ITGB2, which modulates cell adhesion and inflammatory signaling, might also play a role. There may even be a significant interplay among multiple genes in this region, some of which may act synergistically or independently.

The vast majority of models of DS utilize mice. The only known *rat* model was generated by the Herault group at the Institute of Genetics and Molecular and Cellular Biology in Strasbourg, France. In the rat genome, the human chromosome 21 homologous regions are located on two chromosomes, chromosome 11 and chromosome 20. The transgenic Sprague-Dawley Dup(Rno20)Yah rat model of DS, contains the duplication of rat chromosome 20 which, among others, encodes for CBS [24]. Accordingly, in this model, CBS expression was significantly upregulated across various brain regions, including the prefrontal cortex, hippocampus, entorhinal cortex, and basal forebrain [24]. In some of these regions, the upregulation of the truncated, constitutive form of CBS was particularly prominent. CBS was primarily localized to astrocytes, corroborating findings from human DS samples and mouse models discussed earlier. In addition to CBS, increased levels of 3-MST were observed in several brain regions, while CSE expression remained unchanged. For H_2_S degradation enzymes, region-specific alterations were noted. TST expression was reduced in the entorhinal cortex, while ETHE1 expression was slightly elevated in the prefrontal cortex of DS rats, likely as compensatory responses to excess H_2_S. Sulfide quinone reductase (SQOR) expression was unchanged across brain regions [24].

Similar to the mouse model of DS discussed earlier, reactive astrogliosis, a hallmark of DS pathology, was prominently observed in the hippocampus of Dup(Rno20)Yah rats, evidenced by increased astrocytic proliferation and hypertrophy. Immunohistochemical analyses revealed that CBS overexpression was localized predominantly in astrocytes, suggesting a direct link between CBS activity and gliosis. AOAA treatment significantly reduced astrocyte numbers and complexity in the DS hippocampus, indicating that CBS inhibition alleviates gliosis, presumably by modulating H_2_S levels. This was associated with a reduction of the overexpression of CBS in the DS brain [24]. Similar reductions in reactive astrogliosis and CBS expression were also observed in a mouse model of DS in response to pharmacological inhibition of CBS [27]. In the rat model of DS, there was a significant reduction in total CBS protein after treatment with AOAA [24]: as AOAA's primary pharmacological effect is the inhibition of the enzyme's activity (rather than its expression), a reduction in the *expression* of CBS in the rats treated with AOAA is *not* expected. However, if the CBS inhibitor reduces the amount of glia cells in the brain (which contain the majority of CBS protein), the observed effect can be rationalized.

**Fig. 19:**
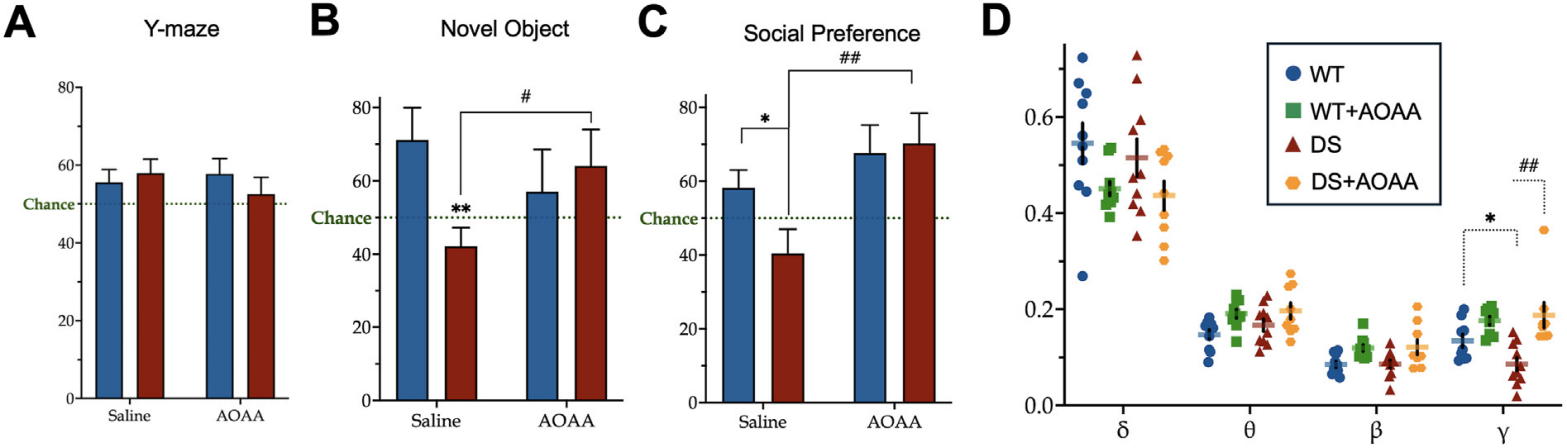
**CBS inhibition restores recognition memory and normal EEG patterns in a rat model of DS**. Wild-type and DS rats with and without treatment with the CBS inhibitor AOAA were assessed in **(A)**: the spontaneous alteration test (T-maze); **(B)**: in the novel object recognition assay and **(C)**: in a preference for social novelty test. The DS rats did not exhibit any impairment in the T-maze test, and AOAA treatment did not affect the responses. However, in the novel object recognition and social novelty tests, DS rats showed significant deficits, which were improved by CBS inhibition. **(D)**: DS is associated with alterations in the electrical wave patterns of the brain; normal patterns are restored by CBS inhibition. Spectra in the basal forebrain are shown. DS rats exhibited significant spectral alterations, with a significant decrease in γ oscillations. AOAA treatment restored γ activity and further stimulates θ and β oscillations. ∗p ​≤ ​0.05. Reproduced by permission from Ref. [24].

The Dup(Rno20)Yah rats exhibited pronounced deficits in recognition memory, as demonstrated by reduced performance in the novel object recognition test [24] (Fig. 19). These impairments were *not* due to differences in locomotion or anxiety levels, as confirmed by comparable open-field test results between DS and control rats. Social recognition memory was also impaired, with DS rats failing to discriminate between novel and familiar conspecifics in a social preference paradigm [24] (Fig. 19). Electrophysiological analyses revealed suppressed gamma oscillations in the entorhinal cortex and basal forebrain of DS rats, accompanied by decreased expression of synaptic proteins such as PSD95 and synaptophysin [24]. These disruptions in synaptic function and brain wave patterns align with the synaptopathy observed in mouse models and human DS brains. Pharmacological inhibition of CBS with AOAA reversed many DS-related deficits in the Dup(Rno20)Yah rats; these effects included a restoration of the EEG spectra [24] (Fig. 19). AOAA treatment of DS rats also improved recognition memory and social preference performance, consistent with its effects in CBS-overexpressing mouse models [24]. At the synaptic level, AOAA normalized the expression of PSD95 and synaptophysin and restored gamma oscillations in affected brain regions, indicating improvements in synaptic integrity and neural network activity [24]. Furthermore, AOAA enhanced mitochondrial function in DS hippocampal tissues *ex vivo*, increasing ATP production [24]. This latter effect is most likely due to alleviation of H_2_S-mediated inhibition of mitochondrial Complex IV. The above findings in the rat model of DS support the hypothesis that CBS and H_2_S overproduction contribute significantly to DS pathology and provide preclinical validation to CBS as a potential experimental therapeutic target.

## Clinical aspects

As detailed in the first section of the current article, multiple lines of clinical evidence show the upregulation of the CBS pathway and the overproduction of H_2_S in human DS. These findings, coupled with the preclinical data with CBS inhibition in DS cells and DS rodent models may be a clinically relevant pathogenetic factor in DS, especially in the context of metabolic and bioenergetic imbalance and impaired neuronal function and neurocognition. The role of CBS as a factor determining cognitive function is also supported by gene-wide association and gene polymorphism studies in the general population and in homocystinuria patients [[219], [220], [221], [222], [223], [224], [225], [226]]. Efforts to correlate homocysteine levels with intellectual disability in DS yielded results that are difficult to interpret. While, generally, DS is associated with lower-than-normal homocysteine levels, within the DS population, individuals with *higher* CBS expression/activity — based on the Kamoun hypothesis — would be expected to manifest with the *more severe* neurocognitive impairment, and the same individuals would also be expected to have the *lowest* plasma homocysteine levels. However, the clinical data indicate that, within the DS population, individuals with relatively *higher* plasma homocysteine levels exhibit more severe cognitive deficits [75,227,228]. The reason for this observation may be that homocysteine, *on its own*, may exert inhibitory effects on neurodevelopment [[229], [230], [231], [232]]. One could envision a bell-shaped dose-response relationship, where aberrantly high CBS activity and H_2_S levels induce metabolic and developmental impairment via mitochondrial inhibition — even in the absence of elevated homocysteine levels, while at the other end of the curve, high homocysteine levels, on their own, also exert adverse effects in individuals with relatively lower CBS activity. The relationship between homocysteine and H_2_S is further complicated by the fact that H_2_S can *protect* against the cytotoxic effect of homocysteine [[233], [234], [235], [236], [237], [238], [239]].

The independent, significant pathogenetic role of elevated H_2_S levels in the CNS is also indirectly supported by clinical data. While elevated H_2_S levels have been implicated in the pathogenesis of multiple CNS diseases — including schizophrenia and amyotrophic lateral sclerosis [[240], [241], [242]] — perhaps the best example is ETHE1 deficiency, a rare, severe disease where an inactivating mutation in ETHE1 protein severely impairs the patients cells' ability to detoxify H_2_S. This rare autosomal recessive condition is known as ethylmalonic encephalopathy. It is characterized by severe neurological dysfunction which includes developmental delay, progressive psychomotor regression, hypotonia, and seizures. The pathological accumulation of H_2_S in the brain, and the consequent disruption of mitochondrial function via inhibition of cytochrome *c* oxidase is considered the primary biochemical foundation of these neurological manifestations [[243], [244], [245], [246], [247]]. Deficiency of SQOR, another enzyme involved in H_2_S degradation, produces a similar neurotoxic picture, both in preclinical models and in patients [248,249]. The clinical picture of ETHE1 and SQOR deficiency lends indirect support to the notion that an elevated brain H_2_S level, on its own (i.e., even in the absence of any other pathobiochemical event) can exert marked, deleterious metabolic and neurological defects in humans.

Although the idea that a single enzyme or pathway, such as CBS may play pathogenetically and experimentally relevant roles in DS is supported by the multiple lines of data overviewed in the previous sections, in the clinical literature the idea that DS is a multi-etiological condition, and not driven by any single biochemical pathway, has been the predominant view in the clinical literature for several decades. Much attention focused on the identification of a so-called "Down syndrome critical region (DSCR)" on a segment near 21q22.3, which does not include the cbs gene [[250], [251], [252], [253]]. However, subsequent studies in mouse and human models, including partial trisomy cases, demonstrated that multiple regions across the chromosome contribute to DS phenotypes and the idea that the DS phenotype results from the combined effects of multiple genes located throughout chromosome 21 — likely interacting in complex manner — rather than the effect of a single critical region in chromosome 21. There are also well documented effects beyond gene dosage (the triplication of genes), related to widespread changes in the regulation of genes encoded by all chromosomes – chromosome 21. These include wide-ranging changes in chromatin structure and transcriptional networks across chromosomes as well as the importance of epigenetic regulation, noncoding RNAs, and genome-wide interactions [46,64,106,254,255].

An obvious – although technically challenging – therapeutic options in DS would be the therapeutic inactivation of the extra chromosome 21. This intervention would not only prevent CBS overexpression, but would exert widespread and pluripotent beneficial effects, particularly if introduced during the early stages of development. Such an approach holds the promise of addressing the root genetic cause of DS. While significant progress has already been made toward the technical feasibility of chromosome inactivation [256], the translational and clinical applicability of this method remains uncertain. Challenges include the potential risks of off-target effects, ethical considerations, and the complexities of delivering such a therapy at the appropriate developmental stage. Consequently, alternative strategies that leverage existing pharmacological knowledge and state-of-the-art drug development methodologies are currently more pragmatic and deserve attention. In this context, the CBS/H_2_S pathway emerges as "driver pathway" in DS and a compelling therapeutic target. Based on the preclinical evidence overviewed in the prior sections, pharmacological inhibition of CBS, the enzyme responsible for the overproduction of H_2_S, represents a preclinically validated strategy for ameliorating neurocognitive deficits in the young-adult to adult period of DS. Whether this approach may also influence the later stages of the condition (DS-associated AD) is unclear at this moment; the potential role of this pathway in chronic neurodegeneration remains to be investigated.

But, how far away are we from a clinically translatable approach to mitigate the "H_2_S burden" in DS? Based on the example of the clinical management of ethylmalonic encephalomyelopathy patients [247,248], dietary restriction of sulfur-containing amino acids, such as methionine and cysteine, along with supplementation of cofactors that support residual sulfur metabolism, such as riboflavin and ubiquinone may be considered. Another approach in ethylmalonic encephalomyelopathy is treatment with metronidazole: this antibiotic reduces colonic bacterial burden, and is expected to reduce H_2_S levels via suppression of H_2_S generation by enteric bacteria [247,248]. However, given the fact that bacterial H_2_S is a relatively minor contributor to systemic H_2_S levels in humans [17,129], the expected efficacy of this approach is low: it is unlikely that it would be able to reduce H_2_S burden in the brain of DS individuals. In fact, the efficacy of these dietary and antibiotic-based approaches is also rather limited in ethylmalonic encephalomyelopathy [223,224].

In 2019 Kamoun proposed the use of H_2_S scavengers, such as sodium nitrite or B12 vitamin derivatives such as hydroxycobalamin or cobinamide [10]. It is unclear if the clinical efficacy of these compounds would be sufficient to exert significant therapeutic effects in DS. Moreover, these compounds would be consumed in the reactions with H_2_S, and high-dose therapy would be necessary. The pharmacological and biochemical characteristics of the currently clinically available cobalamin-based molecules (including their oral bioavailability, plasma half-life, CNS penetration and reaction rates with H_2_S) are suboptimal and would make their clinical translation to DS difficult. Nevertheless, the *concept* of H_2_S neutralization itself should not be dismissed: perhaps some innovative, preferably catalytic H_2_S-degrading nanotechnological or biotechnological approaches may be developed in the future.

A more direct approach would be pharmacological inhibition of the catalytic activity of CBS. In preclinical studies the most commonly used CBS inhibitor is AOAA, a compound that binds to the PLP group in the active site of the enzyme, and is a largely irreversible inhibitor of the enzyme [50,257]. Its pharmacological properties, selectivity and the limited human clinical trial experience with this compound has been overviewed, in detail in 2020 [50]. There are multiple lines of preclinical data with this compound, demonstrating its beneficial effects in DS cells and in mouse and rat models of DS [18,24,25,27] as well as in various other CNS diseases associated with memory impairment and/or neurobehavioral defects [[258], [259], [260]]. There is also a prodrug derivative of AOAA, with improved cell uptake [261]. Nevertheless, the selectivity of this compound — which is known to inhibit several other PLP-dependent enzymes, some with clearly defined physiological roles in the CNS — makes it unattractive as a clinical development candidate. Even if an attempt were to be made to reintroduce AOAA into clinical trials, it would be treated as a “new chemical entity” by the regulatory authorities. This would necessitate a comprehensive set of new safety and toxicology studies, along with GMP-compliant synthesis of the compound.

The green tea polyphenol epigallocatechin-3-gallate (EGCG) has many actions including antioxidant, anti-inflammatory, antibacterial, anticancer, and neuroprotective properties. Multiple pharmacological modes of action underlie these effects, including modulation of inhibition of ROS production and direct scavenging of free radicals. In the context of DS, EGCG primarily viewed as an inhibitor of DYRK1A, a dual-specificity kinase overexpressed in DS and linked to neurodevelopmental and cognitive deficits [262,263]. Green tea extracts containing EGCG have been explored in small-scale clinical trials of DS, largely with negative outcomes [[264], [265], [266]]. In these trials, the actual plasma levels of EGCG have not been measured, and it is unclear if the dosing regimens achieved the required effective concentrations in the DS subjects. Interestingly, EGCG was also found to be a fairly potent of CBS, via binding to a region adjacent to the channel that leads to the enzyme's active site [26]. EGCG, in the concentration range of 0.3–10 ​μM, reduces H_2_S generation in human DS fibroblasts *in vitro* and improves their proliferation [26]. The efficacy of EGCG has not yet been assessed *in vivo* in DS models that contain triplication of the CBS gene. Although significant efforts are being exerted on improving the pharmaceutical properties of EGCG — such as improving its oral bioavailability and prolonging its plasma half-life [267,268] — it is unclear if these efforts will ultimately produce a strong, clinically viable drug formulation suitable for high-quality clinical trials. And — even if the pharmaceutical properties of the molecule are improved — EGCG remails a highly promiscuous molecule with a wide range of pharmacological actions, which include, among others, inhibition of multiple matrix metalloproteinases, inhibition of cyclooxygenase-2 and 5-lipoxygenase, inhibition of ornithine decarboxylase, inhibition of proteasomal activity, inhibition of histone deacetylases, and diverse effects on signaling pathways including signaling pathways associated with EGFR, JAK-STAT, MAPKs, NF-κB and the PI3K-AKT-mTOR [[269], [270], [271], [272], [273]]. While, in theory, a combined inhibitor of Dyrk1 and CBS in DS may be an attractive approach — given that both of these enzymes play important, and mostly non-overlapping pathogenetic mechanisms in DS — the potential utility EGCG-based approaches in a clinical DS setting remains to be further elucidated.

A CBS inhibitor with potential for repurposing, identified through a physical screening campaign on human recombinant CBS [274], is the clinically utilized DOPA decarboxylase inhibitor benserazide. It is not marketed as a standalone drug but is formulated as part of the combination therapy Madopar (benserazide ​+ ​l-DOPA) in many European countries and Canada. Similar to AOAA, benserazide inhibits CBS through a PLP-dependent mechanism and in various cancer models, and produces the type of responses that would be expected from a CBS inhibitor *in vitro* and *in vivo* — although the required concentrations/doses are fairly high [274,275]. Notably, it has been shown to elevate plasma homocysteine levels in humans [276], which is consistent with its inhibitory action on CBS. However, its relatively low potency as a CBS inhibitor, coupled with its poor penetration into the CNS limits its potential utility for DS.

Disulfiram is another potentially repurposable CBS inhibitor. This drug is a well-established medication primarily used in the treatment of chronic alcohol dependence [277]. Its mechanism of action involves the irreversible inhibition of aldehyde dehydrogenase (ALDH), a critical enzyme in alcohol metabolism. However, beyond its primary use in alcohol dependence, disulfiram has additional pharmacological effects due to its ability to chelate metal ions, such as copper and zinc, and inhibit a range of enzymes (dopamine β-hydroxylase, monoamine oxidases, xanthine oxidase, carbonic anhydrase and others) which contributes to its biological activity. Repurposing of disulfiram (or its metabolites, particularly diethyldithiocarbamate) has been proposed to be applied in anticancer and antimicrobial contexts [[277], [278], [279]]. The fact that disulfiram also have actions as a CBS inhibitor discovery emerged from a yeast-based screening [19] and its efficacy to improve CBS-associated neurocognitive impairment was confirmed in CBS-overexpressor mice *in vivo* [19]. It was subsequently demonstrated that disulfiram itself does not directly inhibit CBS. Instead, its metabolite, bis(N,N-diethyldithiocarbamate)-copper(II) complex is responsible for a combined effect which combines an inhibition of CBS activity with scavenging of H_2_S, producing a cell-based inhibition of H_2_S signal and improvement of the proliferation of DS cells in the low micromolar concentration range [280]. A comparable potency for other targets has been deemed suitable to support various repurposing concepts for disulfiram, which culminated in various clinical trials [278,279]. Disulfiram has a high ability to cross the blood-brain barrier [277]. Thus, a repurposing effort and potential clinical trial with disulfiram in DS should is theoretically conceivable, although it is unclear whether the compound's selectivity and safety profile would support long-term administration.

There are several other CBS inhibitors, which are preclinical experimental compounds used to characterize the role of CBS in various conditions, but have not been considered in translational or clinical contexts for DS or for other human conditions. These compounds — including NSC67078, aurintricarboxylic acid, JHU-8555 and many others — are discussed in various specialized review articles [50,281].

In the current review we focused on the CBS pathway alone, and did not discuss the many other pathways that have been implicated in DS-associated functional defects. Nevertheless, one particular pathway should be briefly mentioned, and this is DYRK1A, because a potent inhibitor of this kinase is progressing into clinical trials [282]. There were several attempts to determine if there is a functional interaction between DYRK1A and CBS, and these attempts did not reveal any clear conclusions, although a recent study suggests that suggest that the overexpression of DYRK1A and CBS have additive effects and that CBS expression and/or activity is increased following DYRK1A overexpression [19,283]. If DYRK1 inhibitors and CBS inhibitors affect different, potentially non-overlapping pathways of bioenergetics and neurological dysfunction, future combined therapy with these two agents may be conceivable. Interestingly, molecular docking and dynamics simulations suggested that apigenin and naringenin may act as potential multifunctional inhibitors for the future experimental therapy of DS because they may simultaneously inhibit CBS and DYRK1A [284].

## Translational directions

To translate the CBS inhibitor concept into practice, the identification and clinical development of an adequately selected, novel CBS inhibitor would be ideal. Such a drug would need to meet several critical criteria: it should be selective for CBS to avoid off-target effects, exhibit high potency to achieve therapeutic efficacy, possess oral bioavailability for ease of administration, demonstrate sufficient penetration into the CNS to target the brain — which is typically achieved by relatively low molecular weight, a certain lipophilicity, and minimal interaction with efflux transporters such as P-glycoprotein. An ideal CBS inhibitor would require a favorable safety and tolerability profile — all typical expectations of successful small-molecule drugs targeting neurodegenerative and neurodevelopmental disorders.

A prototypical CBS inhibitor (i.e., a competitive binder at the active site of the enzyme) would likely inhibit all catalytic activities of this enzyme, including those reactions that are H_2_S-generating (e.g., the conversion of cysteine and homocysteine into cystathionine and H_2_S) as well as those that are not (e.g., the condensation of homocysteine with serine to form cystathionine) [50]. In addition to reducing H_2_S generation, a CBS inhibitor would also be expected to increase homocysteine levels, potentially inducing hyperhomocysteinemia. This elevation in homocysteine could have downstream effects on folate and methylation pathways, thereby impacting cellular methylation status and epigenetic regulation. Moreover, the inhibition of CBS may alter the delicate balance of other metabolites within the transsulfuration pathway, such as glutathione and taurine, which are critical for cellular redox homeostasis and detoxification processes. CBS inhibition may also affect methionine, S-adenosyl homocysteine, S-adenosyl methionine and cysteine levels. Thus, while reducing H_2_S levels may confer therapeutic benefits in conditions of H_2_S overproduction in DS, the simultaneous effects of homocysteine and glutathione homeostasis could have some adverse effects, particularly in tissues with high oxidative stress or metabolic demand. This underscores the importance of selectively modulating CBS activity in a manner, perhaps by focusing on the clinical testing of a CBS inhibitor with high CNS uptake, which would then preferentially concentrate in the brain, as opposed to the peripheral tissues. Nevertheless, mild elevations in circulating homocysteine would not be expected to pose a significant cardiovascular risk, and, in fact, measurement of homocysteine plasma levels in a clinical trial could be used to monitor compliance and target engagement.

**Fig. 20:**
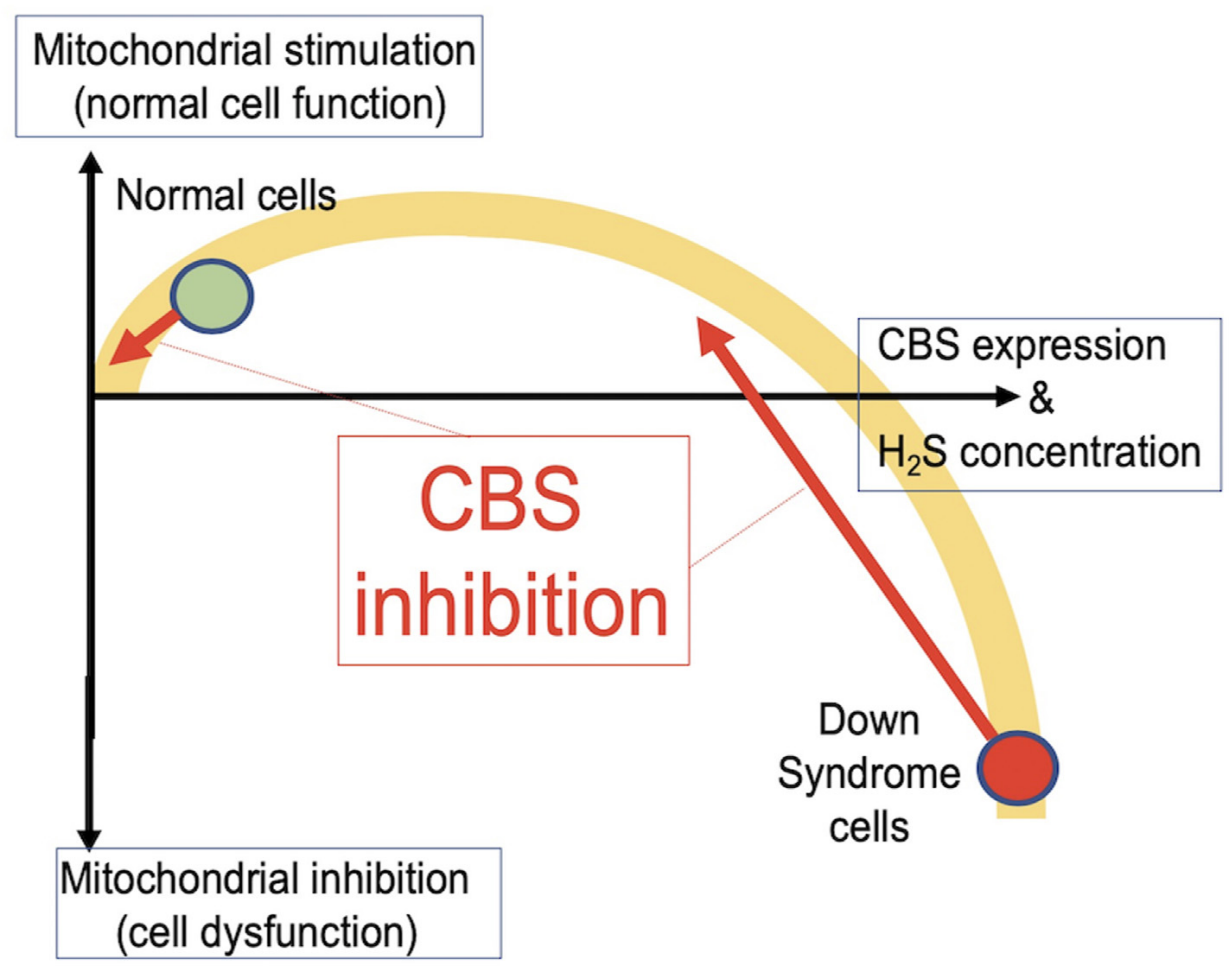
**Role of low and high levels of H_2_S on mitochondrial function: comparison of the bioenergetic profile of control cells and Down syndrome cells**. The x axis denotes increasing expression of CBS and corresponding increasing intracellular concentrations of H_2_S. The y axis denotes stimulation or inhibition of mitochondrial function. In normal cells, the lower endogenous levels of H_2_S do not affect mitochondrial function in a significant manner or exert a slight stimulatory effect. Therefore, inhibition of CBS (red arrow) does not affect this parameter significantly, or may induce a slight inhibitory effect. In DS cells, high intracellular H_2_S levels induce Complex IV blockade and suppress mitochondrial function. Under these conditions, inhibition of H_2_S biosynthesis (red arrow) improves mitochondrial function. The best therapeutic effect of a CBS inhibitor is expected at doses of the inhibitor that restore physiological CBS activity, without excessive over-inhibition of this enzyme. Reproduced by permission from Ref. [18].

One of the advantages of targeting CBS is that therapy would not necessarily need to begin prenatally or during early childhood, which would otherwise introduce substantial regulatory and ethical complexities. Preclinical studies suggest that even short-term treatment in young-adult or adult DS models can yield meaningful benefits in neurocognition and reduce reactive gliosis [24,27]. For instance, in animal models, intermittent treatment regimens—such as two-week courses of the CBS inhibitor AOAA — have demonstrated promising outcomes [24,27]. Translating these findings to clinical practice might involve intermittent therapy schedules, such as 2–3 months of treatment, potentially repeated annually, to sustain cognitive improvements. The dose of the CBS inhibitor would have to be carefully selected, in order to achieve normalization of CBS activity, but to avoid over-inhibition of the target (Fig. 20). This important point is substantiated by data demonstrating the physiological regulatory role of H_2_S in the brain (as discussed in prior sections), as well as by preclinical data with AOAA which shows that neither doses that are too low, nor doses that are too high are efficacious in a murine model of DS [27]. This issue necessitates the careful consideration of biomarkers in order to confirm target engagement and to select the optimal dosing regimen. Markers of H_2_S production (e.g. thiosulfate) or CBS activity (e.g. homocysteine, cystathionine, lanthionine or others) [11,12,285], along with physiological parameters such as EEG spectral analyses—which are often altered in DS [[286], [287], [288], [289], [290]] and have been shown to normalize with CBS inhibition in preclinical studies [24] — could be incorporated into future clinical trials.

The primary endpoint in clinical trials should emphasize neurocognition, a domain of profound clinical importance in DS and one where preclinical evidence with pharmacological CBS inhibition [24,27] or genetic CBS normalization [19] suggests efficacy. Standardized neurocognitive tests could be employed to evaluate memory, attention, and executive function. Additionally, caregiver-reported outcomes and quality-of-life measures could complement these objective assessments to provide a holistic view of therapeutic impact.

To improve the chance of obtaining clinical proof of efficacy, one could consider stratifying participants based on biomarkers indicative of high CBS activity or elevated H_2_S production. This enrichment strategy could enhance the likelihood of detecting a therapeutic effect, particularly in the early phases of drug development. Additionally, emerging preclinical evidence suggests potential sex differences in CBS expression, homocysteine levels and protein persulfidation patterns in DS [27,193,290], which warrants attention in the design and analysis of future trials. Regarding the role of CBS in the context of sex differences in DS models, the only currently available information exists in a mouse model [27], where the higher CBS expression in DS was primarily observed in the female mice, and the increased brain H_2_S levels was also primarily driven by the female subgroup. Moreover, the changes in persulfidated proteins were more pronounced in female mice, and the neurobehavioral differences and the effect of CBS inhibition were also more prominent in the female group. It remains to be determined in future studies if these differences are relevant for other DS models or for human DS pathophysiology and whether this observation should have relevance for future clinical trial designs.

The young-adult DS population could be an ideal target group for initial clinical trials. This demographic balances the need for early intervention to prevent irreversible neurocognitive decline with practical considerations such as consent and compliance.

Given the body of evidence demonstrating the protective role of H_2_S against atherosclerosis, reperfusion injury, cardiomyopathy, hypertension and other cardiovascular diseases [[291], [292], [293], [294], [295], [296]], the question if treatment with CBS inhibitor may increase the risk of cardiovascular events in DS individuals should also be considered. Indeed, it is well documented that individuals with DS tend to be protected from cardiovascular disease and almost never develop hypertension [[297], [298], [299], [300], [301]]: one of the reasons for this protection may be their elevated H_2_S level.

Another line of studies demonstrates that H_2_S, generated by CBS, or 3-MST also plays a pathogenetic role in several types of cancer [[302], [303], [304], [305], [306]]. Given the importance of these processes, coupled with the special oncological status of DS individuals — in essence, protection from many types of solid tumors, but increases in the incidence of several hematologic malignancies [52,79,[307], [308], [309], [310]] — the potential oncological implications of inhibiting CBS in DS individuals should also be considered.

It is unclear at the moment if CBS inhibition may also affect the neuropathology of DS in later stages of the condition. Alzheimer-type neurodegeneration is characteristic in DS, and CBS expression has been detected in DS-associated Alzheimer's [13] (Fig. 6) but, thus far, there are no direct studies to investigate the role of H_2_S or CBS in its pathogenesis specifically in DS-associated AD. In a mouse model of Alzheimer's (male APP/PS mice), no CBS overexpression or H_2_S overproduction was observed, but, rather, a time-dependent decrease was noted [311,312]. Future studies, specifically in DS-associated Alzheimer's models, remain to be conducted to directly investigate the role of the CBS/H_2_S pathway in this condition.

The design and execution of a clinical trial for a CBS inhibitor in DS are undoubtedly complex, but they are no more daunting than the challenges encountered in other trials targeting CNS diseases. Insights from prior Phase Ib and Phase II clinical trials in DS, even those with predominantly negative outcomes [[264], [265], [266],^289^], may provide invaluable guidance for refining trial methodologies, selecting endpoints, and addressing logistical hurdles. For instance, these prior studies often highlighted recruitment challenges and the need for well-defined inclusion criteria to ensure homogeneity in baseline characteristics. By integrating these lessons with robust preclinical evidence and innovative pharmacological strategies, the prospect of developing a CBS inhibitor as a viable therapy for DS will become increasingly attainable.

## Author contributions

CS conceived and wrote the manuscript.

## Declaration of competing interest

The author declares no conflict of interest.
